# Identification of difructose dianhydride I synthase/hydrolase from an oral bacterium establishes a novel glycoside hydrolase family

**DOI:** 10.1016/j.jbc.2021.101324

**Published:** 2021-10-22

**Authors:** Toma Kashima, Kouki Okumura, Akihiro Ishiwata, Machika Kaieda, Tohru Terada, Takatoshi Arakawa, Chihaya Yamada, Kentaro Shimizu, Katsunori Tanaka, Motomitsu Kitaoka, Yukishige Ito, Kiyotaka Fujita, Shinya Fushinobu

**Affiliations:** 1 Department of Biotechnology, The University of Tokyo, Tokyo, Japan; 2 Faculty of Agriculture, Kagoshima University, Kagoshima, Japan; 3 Cluster for Pioneering Research, RIKEN, Saitama, Japan; 4 Collaborative Research Institute for Innovative Microbiology, The University of Tokyo, Tokyo, Japan; 5 Department of Chemical Science and Engineering, Tokyo Institute of Technology, Tokyo, Japan; 6 Faculty of Agriculture, Niigata University, Niigata, Japan; 7 Graduate School of Science, Osaka University, Osaka, Japan

**Keywords:** glycoside hydrolase, enzyme structure, carbohydrate chemistry, fructosyltransferase, crystal structure, D-Ara*f*-α-Me, 1-methyl α-D-arabinofuranoside, DFA, difructose dianhydride, D-Fruf-α-Me, 1-methyl α-D-fructofuranoside, DHL, diheterolevulosan, DUF, domain of unknown function, GH, glycoside hydrolase, HPAEC-PAD, high-performance anion-exchange chromatography with pulsed amperometric detection, MeOH, methanol, *p*NP, *p*-nitrophenyl, *p*NP-α-D-Ara*f*, *p*-nitrophenyl α-D-arabinofuranoside, *p*NP-α-D-Fru*f*, *p*-nitrophenyl α-D-fructofuranoside, TBDPS, tert-butyldiphenylsilane, αFFase1, α-D-fructofuranosidase and difructose dianhydride I synthase/hydrolase from *Bifidobacterium dentium*

## Abstract

Fructooligosaccharides and their anhydrides are widely used as health-promoting foods and prebiotics. Various enzymes acting on β-D-fructofuranosyl linkages of natural fructan polymers have been used to produce functional compounds. However, enzymes that hydrolyze and form α-D-fructofuranosyl linkages have been less studied. Here, we identified the *BBDE_2040* gene product from *Bifidobacterium dentium* (α-D-fructofuranosidase and difructose dianhydride I synthase/hydrolase from *Bifidobacterium dentium* [αFFase1]) as an enzyme with α-D-fructofuranosidase and α-D-arabinofuranosidase activities and an anomer-retaining manner. αFFase1 is not homologous with any known enzymes, suggesting that it is a member of a novel glycoside hydrolase family. When caramelized fructose sugar was incubated with αFFase1, conversions of β-D-Fru*p*-(2→1)-α-D-Fru*f* to α-D-Fru*f*-1,2′:2,1′-β-D-Fru*p* (diheterolevulosan II) and β-D-Fru*f*-(2→1)-α-D-Fru*f* (inulobiose) to α-D-Fru*f*-1,2′:2,1′-β-D-Fru*f* (difructose dianhydride I [DFA I]) were observed. The reaction equilibrium between inulobiose and DFA I was biased toward the latter (1:9) to promote the intramolecular dehydrating condensation reaction. Thus, we named this enzyme DFA I synthase/hydrolase. The crystal structures of αFFase1 in complex with β-D-Fru*f* and β-D-Ara*f* were determined at the resolutions of up to 1.76 Å. Modeling of a DFA I molecule in the active site and mutational analysis also identified critical residues for catalysis and substrate binding. The hexameric structure of αFFase1 revealed the connection of the catalytic pocket to a large internal cavity *via* a channel. Molecular dynamics analysis implied stable binding of DFA I and inulobiose to the active site with surrounding water molecules. Taken together, these results establish DFA I synthase/hydrolase as a member of a new glycoside hydrolase family (GH172).

Inulin-type fructans and fructooligosaccharides, which are composed of β-(2→1) fructosylfructose linkages, are attracting attention as prebiotics in functional foods (1, 2). Recent studies have highlighted the importance of inulin as a dietary fiber in shaping human gut microbes (3, 4). Natural plant fructans, such as inulin and β-(2→6)-linked levan, are all β-linked polymers (5). Enzymes that cleave and/or transfer β-fructofuranosides of fructans, fructooligosaccharides, and sucrose are mainly classified in the glycoside hydrolase (GH) families 32 and 68 (6). Although the enzymes acting on β-fructofuranoside linkages (β-fructofuranosidases and β-fructotransferases) have been extensively studied (7), the knowledge of enzymes acting on α-fructofuranosyl linkages is limited.

Difructose dianhydrides (DFAs) are cyclic disaccharides consisting of 2 fructose units reciprocally linked at their reducing carbons. The disaccharides are generated by the caramelization of fructose-containing foods (8). Commercial sucrose caramel contains a significant proportion of DFAs (∼18%) (9), suggesting that modern humans routinely consume carbohydrates that contain α-fructofuranosyl linkages formed by nonenzymatic dehydration reactions during food baking. Among various DFAs, DFA III (α-D-Fru*f*-1,2′:2,3′-β-D-Fru*f*) and DFA IV (β-D-Fru*f*-2,6′:6,2′-β-D-Fru*f*) have various beneficial effects on human health (10). An industrial production process of DFA III from chicory inulin was established using DFA III-forming inulin fructotransferase (EC 4.2.2.18). DFA III became commercially available in Japan (11, 12). In addition, many microbial enzymes that specifically produce DFA IV or DFA I (α-D-Fru*f*-1,2′:2,1′-β-D-Fru*f*) from levan or inulin have been identified (13). DFA IV-forming levan fructotransferase (EC 4.2.2.16) (14) belongs to GH32 and catalyzes intramolecular transfructosylation, consistent with the general retaining mechanism of GHs (15). In contrast, DFA III-forming inulin fructotransferase catalyzes an inverting intramolecular transfructosylation, resulting in a lyase reaction, and belongs to GH91. GH91 also contains DFA I-forming inulin fructotransferases (EC 4.2.2.17) (16) and DFA III hydrolases (EC 3.2.1.-) (17). The proposed reaction mechanisms of DFA III-forming inulin fructotransferase (18) and DFA III hydrolase (19) are consistent with the standard inverting GH mechanism. GH91 was once reclassified to the polysaccharide lyase family 19 but was returned to GH91 because of its mechanistic similarity to lytic transglycosidases in GH23, GH102, GH103, and GH104 (https://www.cazypedia.org/index.php/Glycoside_Hydrolase_Family_91) (20).

Among the known DFA-producing enzymes, an enzyme catalyzing the formation of DFA I from inulobiose (β-D-Fru*f*-(2→1)-D-Fru) was isolated from the fungus *Aspergillus fumigatus* (21, 22). This enzyme catalyzes the formation of an α-fructofuranosidic bond by reverse hydrolysis (condensation) and has been described as difructose-anhydride synthase (EC 3.2.1.134) by the nomenclature committee of the International Union of Biochemistry and Molecular Biology. However, the gene encoding this enzyme has not been identified.

*Bifidobacterium* is a representative genus of ‘generally recognized as safe’ bacteria among the thousands of species that constitute the human gut microbiota. Probiotic effects have been reported for many bifidobacterial strains (23, 24, 25). Yamamori *et al*. reported that several *Bifidobacterium* species grew by using α-D-Fru*f*-(2→6)-D-Glc (26), suggesting that these bacteria may have α-fructofuranosidase.

In this study, we identified the gene for a difructose-anhydride synthase from the genome of *Bifidobacterium dentium* isolated from human dental caries (27). Based on biochemical characterization, X-ray protein crystallography, and computational analysis, we propose a plausible reaction mechanism for the enzyme and the establishment of a novel GH family.

## Results

### BBDE_2040 is a retaining α-D-fructofuranosidase/α-D-arabinofuranosidase

**Figure 1:**
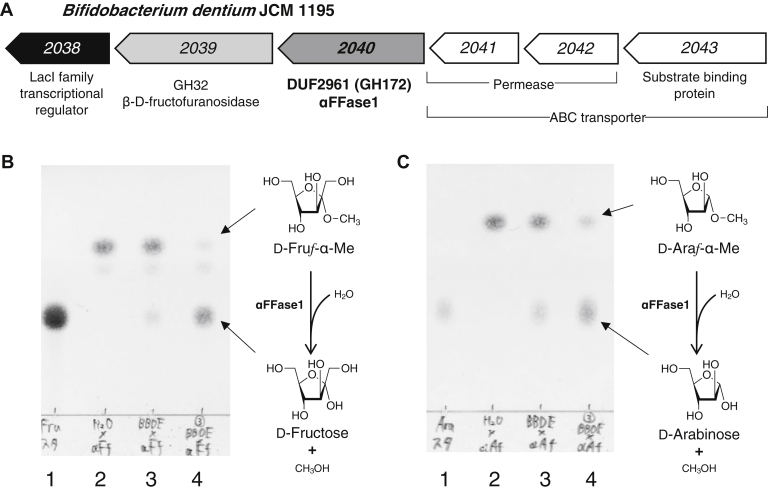
**BBDE_2040 (αFFase1) has α-****D****-fructofuranosidase and α-****D****-arabinofuranosidase activities.***A*, organization of *BBDE_2040* and surrounding genes in the genome of *B. dentium* JCM 1195. The numbers correspond to the locus tag (BBDE_XXXX). *B*, activity of αFFase1 toward D-Fru*f*-α-Me. The substrate (2 mM) was incubated in 50 mM Na acetate (pH 6.0) with purified αFFase1 (12.5 μg/ml) for 100 min (lane 3) or 3 days (lane 4) at 37 °C. Lane 1 contains D-fructose, and lane 2 contains D-Fru*f*-α-Me. *C*, activity of αFFase1 toward D-Ara*f*-α-Me. The substrate (2 mM) was incubated in 50 mM Na acetate (pH 6.0) with purified αFFase1 (12.5 μg/ml) for 100 min (lane 3) or 3 days (lane 4) at 37 °C. Lane 1 contains D-arabinose, and lane 2 contains D-Ara*f*-α-Me. D-Araf-α-Me, 1-methyl α-D-arabinofuranoside; D-Fruf-α-Me, 1-methyl α-D-fructofuranoside; αFFase1, α-D-fructofuranosidase and difructose dianhydride I synthase/hydrolase from *Bifidobacterium dentium*.

In the genome of *B. dentium* JCM 1195, we found an operon consisting of genes encoding a LacI family transcriptional regulator, a putative GH32 β-D-fructofuranosidase, a hypothetical protein (locus tag BBDE_2040 or BBDE_RS10350) that belongs to a domain of unknown function (DUF) 2961, and a set of putative ABC transporter proteins (Fig. 1*A*). To investigate the *in vitro* function of the BBDE_2040 protein, we heterologously produced the C-terminal His_6_-tagged BBDE_2040 protein in *Escherichia coli* and purified it to homogeneity. As the model substrates to study the function of BBDE_2040, 1-methyl α-D-fructofuranoside (D-Fru*f*-α-Me), 1-methyl α-D-arabinofuranoside (D-Ara*f*-α-Me), *p*-nitrophenyl α-D-fructofuranoside (*p*NP-α-D-Fru*f*), and *p*-nitrophenyl α-D-arabinofuranoside (*p*NP-α-D-Ara*f*) were synthesized. When D-Fru*f*-α-Me and D-Ara*f*-α-Me were incubated with BBDE_2040, spots of D-fructose and D-arabinose appeared on TLC (Fig. 1, *B* and *C*), showing that BBDE_2040 has α-D-fructofuranosidase/α-D-arabinofuranosidase activities. We designate the BBDE_2040 protein as α-D-fructofuranosidase and difructose dianhydride I synthase/hydrolase from *Bifidobacterium dentium* (αFFase1) in this study because the natural substrates of this enzyme are supposed to be α-D-fructofuranosyl compounds (described below). *p*NP-α-D-Fru*f* was unstable in aqueous solution (Fig. S1, *A* and *B*), and the activity of αFFase1 was apparently lower for *p*NP-α-D-Fru*f* than for *p*NP-α-D-Ara*f* (Fig. S1*B*). Therefore, *p*NP-α-D-Ara*f* was used as the standard substrate for the enzymatic characterization. The optimal pH and apparent optimal temperature were 5.5 and 50 °C, respectively (Fig. S2, *A* and *B*). The *K*_m_ and *k*_cat_ values toward *p*NP-α-D-Ara*f* were 2.71 ± 0.21 mM and 127.5 ± 4.0 s^−1^, respectively, at pH 6.0 and 37 °C (Fig. S2*C*).

**Figure 2:**
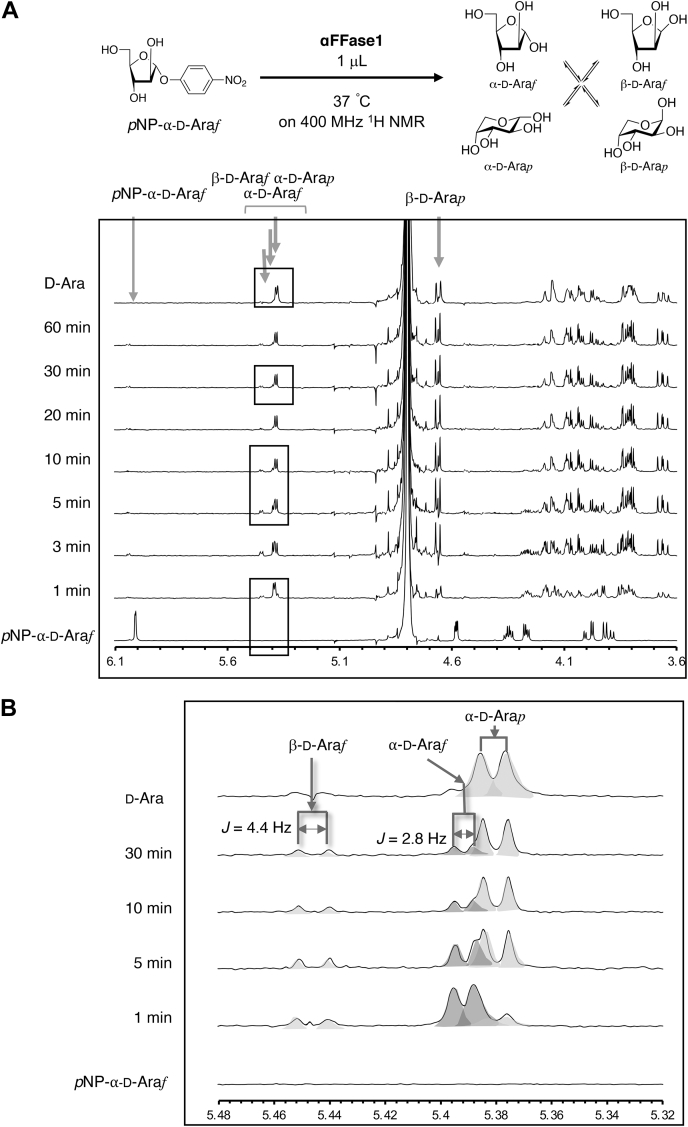
**αFFase1 is an anomer-retaining enzyme.***A*, ^1^H NMR spectra monitoring the activity toward *p*NP-α-D-Ara*f* in D_2_O referenced at DOH. *B*, the enlarged view of the area boxed in panel *A* referenced at H1 of α-D-Ara*p*. The characteristic chemical shifts and *J*-coupling constants at anomeric C1-H of *p*NP-α-D-Ara*f*, β-D-Ara*f*, α-D-Ara*f*, α-D-Ara*p*, and β-D-Ara*p* are indicated by *arrows*. αFFase1, α-D-fructofuranosidase and difructose dianhydride I synthase/hydrolase from *Bifidobacterium dentium*; *p*NP-α-D-Ara*f*, *p*-nitrophenyl α-D-arabinofuranoside.

The stereochemistry of the glycosidic bond hydrolysis was monitored by ^1^H NMR (Figs. 2 and S3). The anomeric hydrogen signal of *p*NP-α-D-Ara*f* (doublet at 6.01 ppm, ^3^*J*_H1–H2_ = 1.2 Hz) disappeared within 1 min. A signal of H-1 of α-D-Ara*f* (doublet at 5.39 ppm*,* ^3^*J*_H1–H2_ = 2.8 Hz) first appeared as the major initial furanoside concomitant with a much weaker H-1 signal of β-D-Ara*f* (doublet at 5.45 ppm, ^3^*J*_H1–H2_ = 4.4 Hz). The anomeric H-1 signals of pyranoses, such as α-D-Ara*p* (doublet at 5.38 ppm, ^3^*J*_H1–H2_ = 3.6 Hz) and β-D-Ara*p* (doublet at 4.66 ppm, ^3^*J*_H1–H2_ = 8.0 Hz), subsequently appeared and clearly increased as more stable forms because of mutarotation under the equilibrium conditions within 30 min. This result indicates that αFFase1 is an anomer-retaining GH.

### αFFase1 catalyzes intramolecular dehydration of fructose disaccharides in caramelized sugars

**Figure 3:**
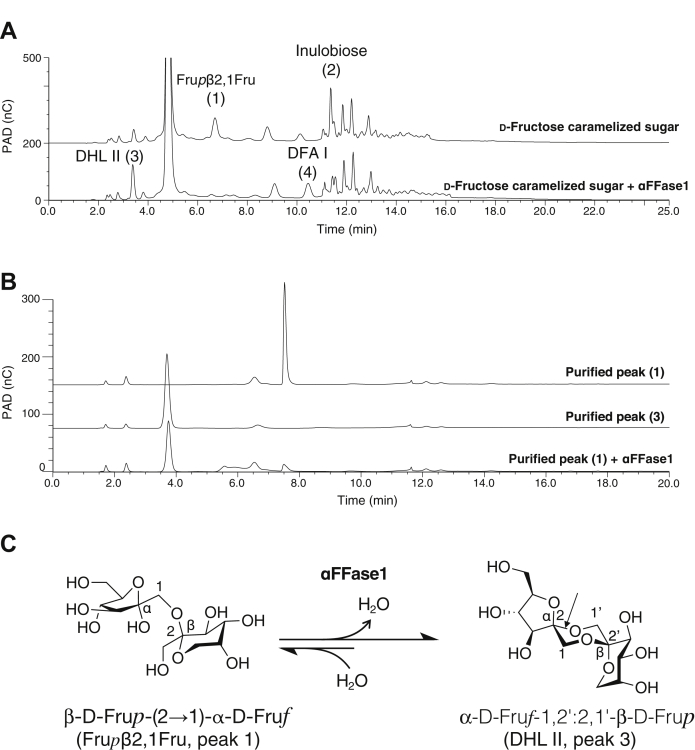
**Activity of αFFase1 toward caramelized sugar made from****D****-fructose.***A*, HPAEC-PAD chromatogram of caramelized sugar alone (*top*) and caramelized sugar treated with αFFase1 overnight at 37 °C (*bottom*). The four peaks that exhibited significant changes by the enzyme treatment are indicated. *B*, HPAEC-PAD chromatogram of the purified peak 1 (*top*), purified peak 3 (*middle*), and peak 1 treated with αFFase1 overnight at 37 °C (*bottom*). *C*, the scheme of the deduced reaction catalyzed by αFFase1 for the conversion from β-D-Fru*p*-(2→1)-α-D-Fru*f* (Fru*p*β2,1Fru) in peak 1 to α-D-Fru*f*-1,2′:2,1′-β-D-Fru*p* (DHL II) in peak 3. The scissile α-D-Fru*f* glycosidic bond in DHL II during the reverse hydrolysis reaction is indicated by an *arrow*. DHL II, diheterolevulosan II; HPAEC-PAD, high-performance anion-exchange chromatography with pulsed amperometric detection; αFFase1, α-D-fructofuranosidase and difructose dianhydride I synthase/hydrolase from *Bifidobacterium dentium*.

Because *B. dentium* colonizes the human oral cavity and intestine (28), we hypothesized that αFFase1 acts on α-D-fructofuranosidic bonds present in caramelized sugars. Figure 3*A* shows the results of high-performance anion-exchange chromatography with pulsed amperometric detection (HPAEC-PAD) analysis of the caramelized D-fructose prepared by heat treatment at 130 °C for 120 min. After incubation with αFFase1, two compounds with retention times at 6.7 and 11.5 min (indicated as peaks 1 and 2) decreased and two compounds at 3.5 and 10.5 min (peaks 3 and 4) increased. Similar peak shifts from peaks 1 and 2 to peaks 3 and 4 were observed for the incubation of the caramelized D-fructose and D-glucose mixture with αFFase1 (Fig. S4). When purified peak 1 was incubated with αFFase1, conversion to peak 3 was observed (Fig. 3*B*). After the overnight reaction, the molar ratio of peaks 1 and 3 was 11.6:88.4. MALDI TOF-MS analysis of these peaks and their peracetylated samples suggested that peaks 1 and 3 were a fructose disaccharide and its anhydride, respectively (Fig. S5). NMR analysis of peak 3 and its peracetylated sample identified this compound as α-D-Fru*f*-1,2′:2,1′-β-D-Fru*p* (Figs. S6 and S7, Tables 1 and S1). It was termed diheterolevulosan (DHL) II (8). The NMR spectrum of the peak 1 sample indicated that this compound is a mixture of β-D-Fru*p*-(2→1)-α/β-D-Fru*f*/*p* (Fru*p*β2,1Fru; Fig. S8). These results suggest that the β-D-Fru*p*-(2→1)-α-D-Fru*f* form of Fru*p*β2,1Fru was converted to DHL II by the catalysis of αFFase1 (Fig. 3*C*).

**Table 1:** Chemical shifts and *J* coupling constants of peak 3 by ^1^H and ^13^C NMR analyses

Assignments	^1^H	^3^*J* Hz	^13^C	Key HMBC
Fru*p*				
1	3.66	d, 12.4	61.23	→ 102.35 (Fru*f*-C2), 95.72 (Fru*p*-C2)
	3.97	d, 12.4		→ 102.35 (Fru*f*-C2)
2	-	-	95.72	
3	3.56	d, 10.0	68.55	
4	3.90	dd, 10.0, 3.6	69.01	
5	3.99	M	69.01	
6	3.72	d, 12.8	63.54	→ 95.72 (Fru*p*-C2)
	3.84	d, 12.8		→ 95.72 (Fru*p*-C2)
Fru*f*				
1	3.50	d, 12.4	61.55	→ 102.35 (Fru*f*-C2), 95.72 (Fru*p*-C2)
	4.29	d, 12.4		→ 102.35 (Fru*f*-C2), 95.72 (Fru*p*-C2)
2	-	-	102.35	
3	3.99	M	81.93	
4	3.94	d, 5.6, 2.0	77.84	
5	4.01	M	83.63	→ 102.35 (Fru*f*-C2)
6	3.73	dd, 12.4, 6.0	61.23	
	3.85	dd, 12.4, 6.0		

Abbreviation: HMBC, heteronuclear multiple bond correlation.

### αFFase1 catalyzes the condensation of inulobiose to DFA I

Because DHL II (α-D-Fru*f*-1,2′:2,1′-β-D-Fru*p*) and DFA I (α-D-Fru*f*-1,2′:2,1′-β-D-Fru*f*) are structurally similar, we presumed that the conversion from peak 2 to peak 4 of the caramelized sugar was that from inulobiose (β-D-Fru*f*-(2→1)-α-D-Fru*f*) to DFA I. When inulobiose was incubated with αFFase1, DFA I was produced (Figs. S9*A* and 4*A*). The reactions of αFFase1 between inulobiose and DFA I were confirmed by ^1^H NMR (Fig. S10*A*) in both directions. The ^1^H and ^13^C NMR chemical shift data of the DFA I sample produced from inulobiose (Fig. S10*B*) were identical to those previously reported (29). Especially, the ^1^*J*_H3–H4_ (2.4 Hz) and ^13^C chemical shifts of C3 at 82.1 ppm unambiguously indicated that α-Fru*f* structure was formed from inulobiose. When the reaction was monitored by HPAEC-PAD using inulobiose or DFA I as a substrate, the reactions reached an equilibrium ratio of DFA I-inulobiose = 89.1:10.9 (Figs. S11 and 4*B*). Matsuyama and Tanaka also reported that the enzyme from *A. fumigatus* resulted in a similar equilibrium biased to DFA I (22). Kinetic analysis of αFFase1 toward inulobiose and DFA I exhibited linear *S*-*v* relationships up to a range of measurable substrate concentrations (Fig. S12). The *k*_cat_/*K*_m_ values estimated from these plots were 0.813 ± 0.016 mM^−1^s^−1^ for inulobiose and 0.0378 ± 0.0032 mM^−1^s^−1^ for DFA I.

**Figure 4:**
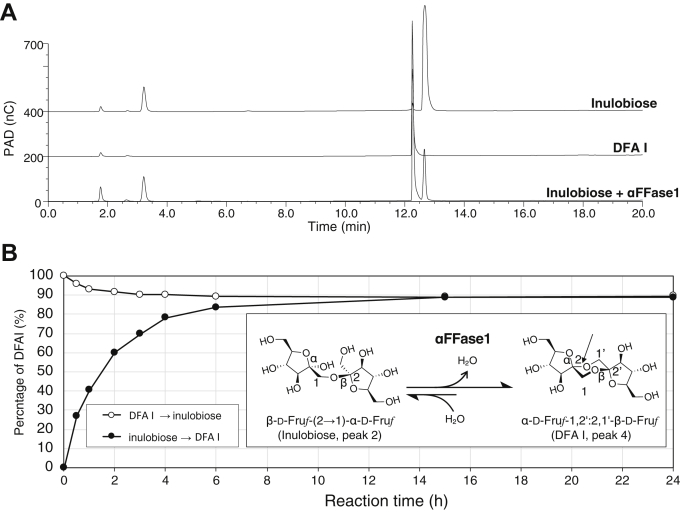
**Activity of αFFase1 toward inulobiose and DFA I.***A*, HPAEC-PAD chromatogram of inulobiose standard (*top*), DFA I standard (*middle*), and inulobiose treated with αFFase1 overnight at 37 °C (*bottom*). *B*, time course of the reaction of αFFase1. DFA I (10 mM) or inulobiose (10 mM) was incubated in 50 mM Na phosphate (pH 6.0) with purified αFFase1 (0.78 μg/ml) at 37 °C. The relative concentrations of DFA I during reactions toward DFA I (*open circle*) or inulobiose (*closed circle*) are shown. The reaction scheme between inulobiose and DFA I is shown in the inset. DFA I, difructose-dianhydride I; HPAEC-PAD, high-performance anion-exchange chromatography with pulsed amperometric detection; αFFase1, α-D-fructofuranosidase and difructose dianhydride I synthase/hydrolase from *Bifidobacterium dentium*.

αFFase1 did not catalyze any reactions when DFA III or inulin was used as a substrate (Fig. S9*B*), and there were no significant peak changes in the reaction of caramelized sugars other than those of the four peaks (Figs. 3*A* and S4). These results indicate that αFFase1 specifically catalyzes the reversible hydrolysis of the intramolecular α-(2→1′)-D-fructofuranosidic bond of DFA I and DHL II. Because of the significant bias of equilibrium toward DFA synthesis, we propose a common name difructose dianhydride I synthase/hydrolase for this enzyme (EC 3.2.1.134).

### Crystal structure of αFFase1

**Figure 5:**
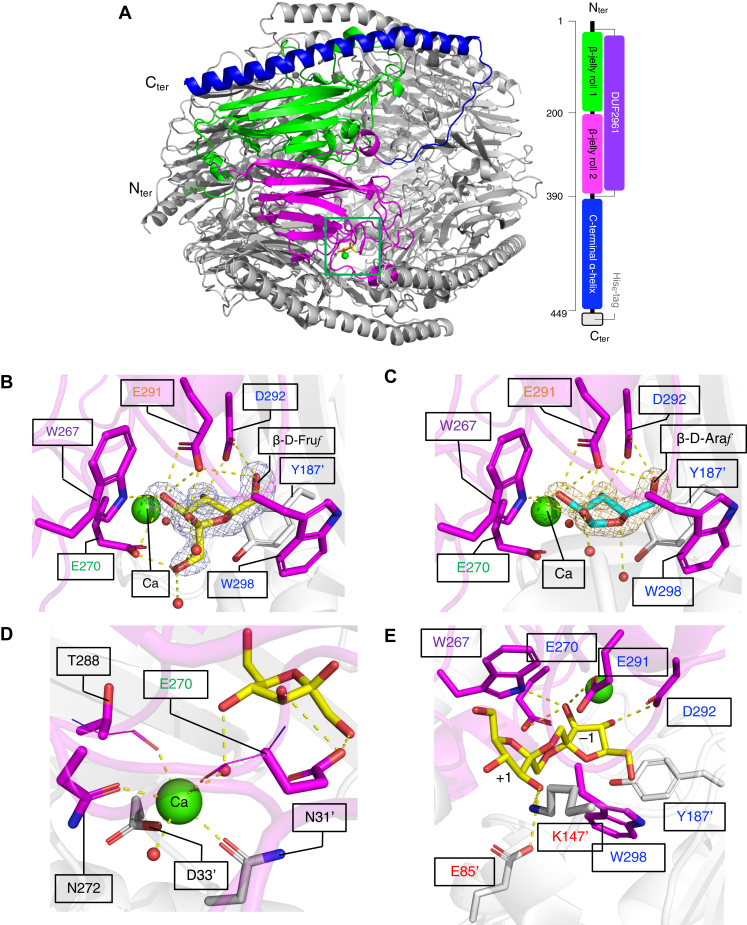
**Crystal structure of αFFase1.***A*, hexameric structure of αFFase1. One protomer is colored to show the β-jelly roll 1 (*green*), β-jelly roll 2 (*magenta*) domains, and the C-terminal α-helix (*blue*). A calcium ion and β-D-Fru*f* are shown in the active site (*green box*) as a *green sphere* and *yellow sticks*, respectively. The domain architecture is shown on the right. *B* and *C*, active site structures of the complex with β-D-Fru*f* (*B*, *yellow sticks*) and β-D-Ara*f* (*C*, *cyan sticks*). The polder maps of β-D-Fru*f* (7.0 σ) and β-D-Ara*f* (8.0 σ) are shown as *blue* or *goldmesh*. E291 and E270 are suggested to be the catalytic nucleophile and acid/base catalyst residues, respectively. *D*, calcium-binding site of the β-D-Fru*f* complex. The main chain groups involved in the calcium coordination are shown as *thin sticks*. The main-chain carbonyl of the catalytic acid/base residue (E270) is involved in the calcium coordination. *E*, modeled DFA I molecule in the active site. In panels *B*–*E*, protein residues from chain A and a neighboring subunit are shown as *magenta* and *white sticks*, respectively. The protein residues constituting the −1 and +1 subsites are indicated by *blue* and *red* characters, respectively. W267, which forms both −1 and +1 subsites, is indicated by *purple* characters. E291 (nucleophile) and E270 (acid/base catalyst) are indicated by *orange* and *green* characters, respectively. DFA I, difructose-dianhydride I; αFFase1, α-D-fructofuranosidase and difructose dianhydride I synthase/hydrolase from *Bifidobacterium dentium*.

The crystal structure of αFFase1 was determined by molecular replacement using the structure of the hypothetical protein BACUNI_00161 (PDB ID: 4KQ7, 36% sequence identity) from *Bacteroides uniformis* ATCC 8492 as a search model. The ligand-free structure was determined at 1.96 Å resolution (Table S2). The asymmetric unit contained a hexamer with *D*_3_ dihedral symmetry. The hexameric structure corresponded to the possible biological assembly that was implied by size-exclusion chromatography and the PISA server (30). The six chains in the asymmetric unit have virtually identical structures, as the average RMSD and their SD for the Cα atoms between all chain pairs are 0.15 ± 0.03 Å (maximum = 0.19 Å in 15 pairs). We will mainly describe chain A, unless otherwise noted. Each monomer consists of β-jelly roll 1 (residues 3–200, green), β-jelly roll 2 (201–396, magenta), and a 51-amino acid–long α-helix at the C terminus (397–448, blue), englobing the whole structure (Fig. 5*A*). The two β-jelly roll domains correspond to CATH superfamily 2.60.120.1390 (protein of unknown function DUF2961) (31). A Dali structural similarity search (32) of the whole monomer showed that αFFase1 is similar to the hypothetical protein BACUNI_00161, which is used as a template for molecular replacement (Table S3). Several capsid proteins of phages showed weak structural similarities (Z score >8). Dali searches of each domain indicated that none of the β-jelly roll folds showed structural similarity to GHs or carbohydrate-binding modules with a Z score higher than 8.

The structures of αFFase1 complexed with D-fructose and D-arabinose were determined at a resolution of 1.76 and 1.87 Å, respectively (Table S2). In the active sites of all six chains, β-Fru*f* and β-Ara*f* were observed in similar conformations at the expected −1 subsite (Fig. 5, *B* and *C*). The furanose ring of β-Fru*f* and β-Ara*f* adopts *E*_5_ (*P* ∼ 35°) and ^3^*T*_4_ (*P* ∼ 54°) conformations, respectively (Table S4), as analyzed by the Altona–Sundaralingam phase angle parameter (33). These conformations correspond to the “northeast” conformation of the pseudorotational map. The fructose and arabinose furanosides in crystal structures usually take the northern hemisphere conformations (34), and a molecular mechanics study indicated that α- and β-Fru*f* are stable in the northeast and northwest conformations, respectively (35). The active site of αFFase1 was located at the interface of the two protomers (Fig. 5*A*, green box). The sugar ligands are surrounded by Trp267, Glu270, Glu291, Asp292, Trp298, and Tyr187′ (prime indicates that this residue is from the neighboring protomer; Fig. 5, *B* and *C*). Notably, Glu291 and Glu270 are positioned close to the anomeric C2 atom from the β and α configuration sides, respectively. Therefore, Glu291 and Glu270 are suggested to be nucleophile and acid/base catalyst residues, respectively.

A metal ion was observed close to the active site (Fig. 5*D*, green sphere), although the buffers used during protein purification and crystallization contained no divalent metal ions. The metal ion in the crystal structure was assigned as Ca^2+^ based on the analysis using the CheckMyMetal server (36). The Ca^2+^ is coordinated by the side chains of Asn31′, Asp33′, and Asn272, the main chain carbonyl of Glu270 and Thr288, and two water molecules. One of the two coordinating water molecules forms a hydrogen bond with the C4 hydroxy group of Fru*f* (C3 hydroxy of Ara*f*). Treatment with excess amount of EDTA did not inhibit the activity of αFFase1 (Fig. S13*A*). The effects of divalent metal ions showed that Cu^2+^ and Zn^2+^ inhibited the activity, whereas other metal ions including Ca^2+^ did not (Fig. S13*B*).

### Analysis of active site residues by substrate modeling and site-directed mutagenesis

Extensive attempts at cocrystallization and soaking experiments with DFA I and inulobiose using the WT enzyme and mutants at the putative catalytic residues (Glu270 and Glu291) did not reveal electron densities in the active site. Therefore, a model of DFA I was placed in the active site of αFFase1 (Fig. 5*E*). In addition to Trp267, Glu85′ and Lys147′ seemed to constitute the +1 subsite.

To validate the possible roles of amino acid residues in the active site, the activities of single substitution mutants of these residues toward *p*NP-α-D-Ara*f* and inulobiose were measured (Table 2). Concerning the catalytic residues, the E270A and E291Q mutants showed significantly decreased activities. Most mutants at the −1 subsite also showed very low activities for both the substrates. For the mutants at Tyr187, a conservative substitution with phenylalanine (Y187F) retained their activities, whereas the alanine substitution (Y187A) was destructive. W298A showed relatively higher activities, probably because the side chain of Trp298 is not directly involved in the formation of the −1 subsite. The mutants at subsite +1 showed noteworthy differences in their activities toward the two substrates. All three mutants (E85A, E85Q, and K147A) showed almost full activity toward *p*NP-α-D-Ara*f*, while they showed <5% activity toward inulobiose. This may reflect that these residues support the correct binding of one fructose moiety of the difructose substrate at the +1 subsite.

**Table 2:** Relative activity of αFFase1 mutants toward *p*NP-α-D-Ara*f* and inulobiose

Mutant	Subsite	Relative activity (%)^a^	
		*p*NP-α-D-Ara*f*	Inulobiose
E85A	+1	97.7 ± 2.0	0.5 ± 0.0
E85Q	+1	100 ± 2.7	3.0 ± 0.0
K147A	+1	94.1 ± 2.9	4.8 ± 0.1
W267A	−1, +1	n.p.	n.p.
Y187A	−1	0.7 ± 0.0	3.2 ± 0.1
Y187F	−1	90.3 ± 2.6	30.7 ± 0.4
E270A	−1	0.6 ± 0.0	0.1 ± 0.0
E270Q	−1	n.p.	n.p.
E291A	−1	n.p.	n.p.
E291Q	−1	0.8 ± 0.0	2.2 ± 1.0
D292A	−1	1.2 ± 0.0	1.3 ± 0.0
D292N	−1	1.4 ± 0.4	0.1 ± 0.0
W298A	−1	16.5 ± 0.6	4.1 ± 0.1

Abbreviation: n.p., not produced in *E. coli*.

The activity toward 2.5 mM for *p*NP-α-D-Ara*f* or 10 mM for inulobiose at pH 6.0 and 37 °C was measured. See Experimental procedures for detailed assay condition.

a Relative activity compared with the WT enzyme (100%).

### Internal cavity of the αFFase1 hexamer and molecular dynamics simulation

**Figure 6:**
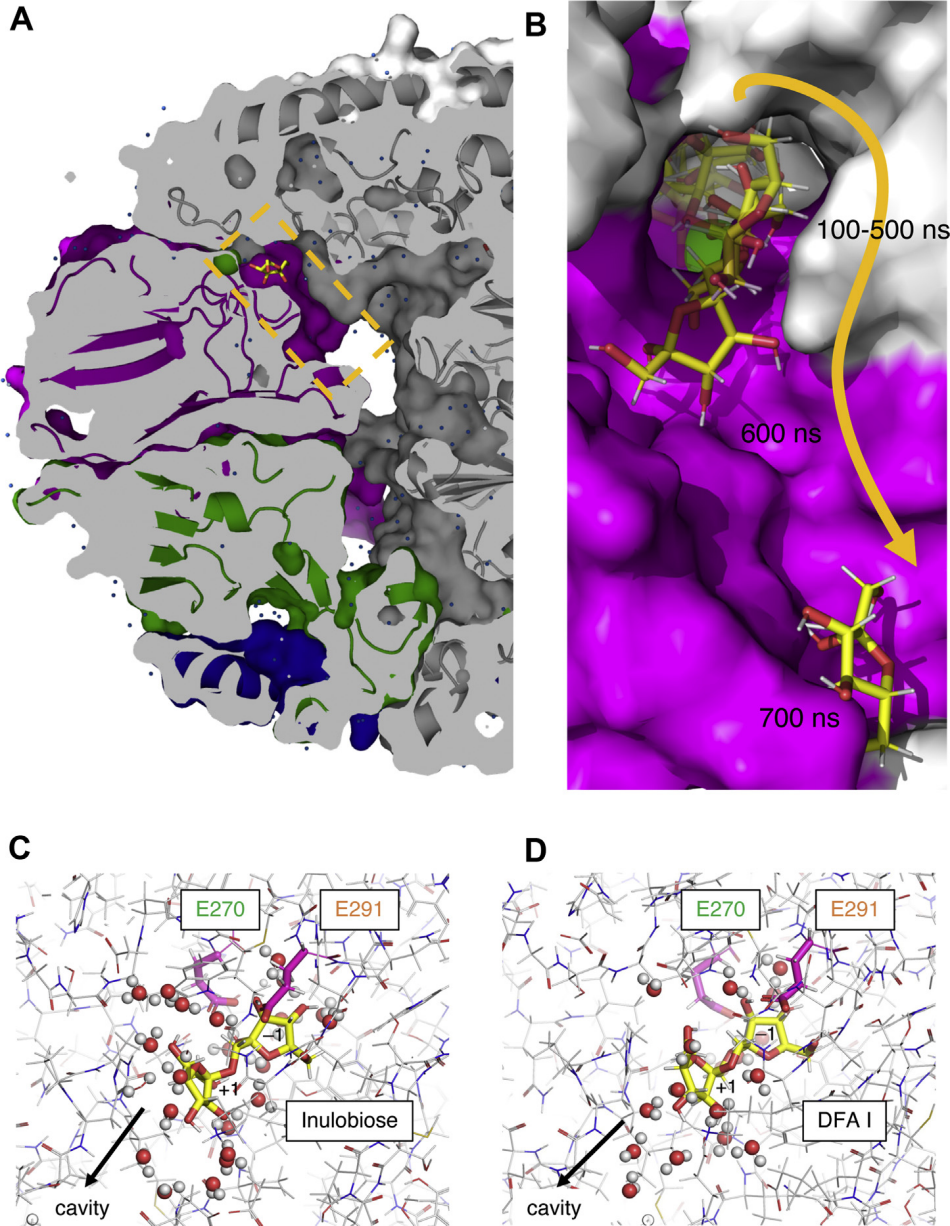
**Active site pocket inside the hexamer and MD analysis.***A*, a cross-sectional view of the active site in complex with β-D-Fru*f*. The channel to the internal cavity is boxed by an *orange dashed line*. *B*, slippage of β-D-Fru*f* from the active site of subunit C during an MD run. The snapshots at 100 to 700 ns are shown. *C* and *D*, snapshots of the active site of subunit A of inulobiose (*C*) and subunit B of DFA I (*D*) at 1 μs. The water molecules within 5 Å are shown as *spheres*. The side chains of the catalytic residues are shown as *magenta sticks*. DFA I, difructose-dianhydride I; MD, molecular dynamics.

Figure 6*A* shows a cross-sectional view of the β-D-Fru*f* complex structure of the αFFase1 hexamer. The active site is located inside the molecule, and there is a channel to a large internal cavity. To investigate the dynamic features of αFFase1, molecular dynamics (MD) simulations starting from the β-D-Fru*f* complex structure and modeled complex structures with inulobiose and DFA I were performed for 1 μs. The whole hexamer of chains A to F was used for the simulation. The ligand in each active site was monitored. β-D-Fru*f* monosaccharides in the active sites of chains A, B, D, E, and F moved back and forth between −1 and +1 subsites, in which approximate distances between the anomeric C2 atom and the nucleophile were 3.5 and 5.0 Å, respectively (Fig. S14*A*). A β-D-Fru*f* molecule in chain C slipped out from the active site during 400 to 700 ns through the channel to the internal cavity (Fig. 6*B*). Thus, the average RMSD of the ligand in chain C during the entire MD run was significantly higher than that of the other chains (Table S5). Inulobiose molecules in chains F and D moved after 250 and 930 ns, respectively, but those in the other chains remained in the active site (Fig. S14*B*). Similarly, DFA I in chains C, E, and A moved after 320, 550, and 850 ns, respectively, but those in the other chains remained at the canonical position (Fig. S14*C*). The snapshots of inulobiose in chain A and DFA I in chain D are shown in Figure 6, *C* and *D*, and their movies are shown in Movies S1 and S2. The MD simulations figured out that many water molecules surrounded each of the ligand molecule. In average, 7 to 9 water molecules were present within 3.5 Å distance to the ligand (Table S5).

## Discussion

### Reaction mechanism

**Figure 7:**
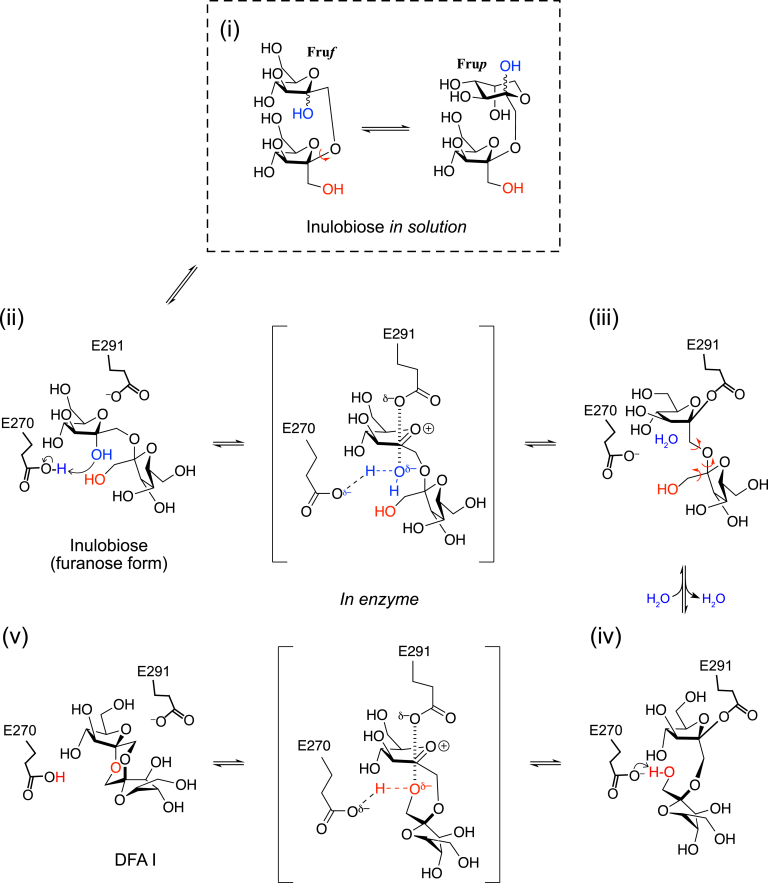
**Proposed reaction mechanism of αFFase1 from inulobiose to DFA I.** Conformational changes of the ligand (bond rotation shown by *red arrows*) are required to fit into the active site and for the nucleophilic attack in the deglycosylation step. DFA I, difructose-dianhydride I; αFFase1, α-D-fructofuranosidase and difructose dianhydride I synthase/hydrolase from Bifidobacterium dentium.

Based on the collective results, we propose a reaction mechanism for αFFase1 in the conversion of inulobiose to DFA I (Fig. 7). (i) The reducing end sugar of inulobiose changes its furanose/pyranose and α/β forms in the internal cavity of αFFase1 as well as in solution because of mutarotation. (ii) The active site of αFFase1 selectively accommodates the α-furanose form in the −1 subsite, as shown in Figure 5. The +1 subsite can also accommodate the pyranose moiety of Fru*p*β2,1Fru (Fig. 3*C*). Glu270 is located at an appropriate position for proton donation (general acid catalysis) to the O2 hydroxy group of α-Fru*f* at the −1 subsite, and Glu291 is placed at a position working as a nucleophile to the anomeric carbon (C2). After this step, the O2 hydroxy group is released from the substrate as a water molecule. (iii) Rotations of the glycosidic bond and the C1–C2 bond of the +1-subsite sugar are required for the intramolecular transfer reaction to occur. (iv) When the C1 hydroxy group of fructose at the +1 subsite is appropriately positioned for proton acceptance (general base catalysis) by Glu270, deglycosylation of Glu291 is facilitated. (v) After the reaction at the active site, DFA I is released through the channel to the inner cavity of the hexamer of αFFase1 (Fig. 6, *A* and *B*). The opposite reaction from DFA I (v) to inulobiose (i) is expected to be similar to the standard retaining reaction mechanism of GHs (https://www.cazypedia.org/index.php/Glycoside_hydrolases) (20). The forward and reverse reactions between DFA I and inulobiose involve water release (condensation) and incorporation (hydrolysis), respectively. The reactions in both directions can occur in the active site pocket because MD analysis indicated that the substrate was always surrounded by water molecules (Fig. 6, *C* and *D*, and Table S5). A Ca^2+^ supports the substrate binding of αFFase1 *via* a water-mediated interaction (Fig. 5*D*). For GHs, Ca^2+^ often contribute to protein stabilization, as exemplified by GH13 α-amylases (37). Several GHs use a Ca^2+^ for substrate recognition: for example, GH43 α-L-arabinanase (38), GH62 α-L-arabinofuranosidase (39), GH47 and GH92 α-mannosidases (40, 41), GH97 α-glucosidase (42), and GH129 α-*N*-acetylgalactosaminidase (43).

### Establishment of a novel GH family

In this study, we identified the bacterial gene of DFA I synthase/hydrolase, whose existence in fungi was reported in previous studies (21, 22). The enzymes producing DFA III and DFA I from inulin (DFA-III- and DFA-I-forming inulin fructotransferases) and DFA III hydrolase have been classified into a single anomer-inverting GH family (GH91). Although DFA III hydrolase apparently cleaves the α-Fru*f* bond of DFA III, the reaction product is inulobiose with β-Fru*f* at the reducing end. The GH91 family of enzymes act on the β-Fru*f* linkages with the lytic transglycosidase-like mechanism (18, 19). Therefore, αFFase1 (DFA I synthase/hydrolase) is a unique anomer-retaining α-fructofuranosidase whose reaction is distinct from the lyase-like inverting GH91 enzymes and DFA IV-forming levan fructotransferases in GH32.

αFFase1 belongs to the DUF2961 (PF11175) family in the Pfam database (44). Although one crystal structure of a hypothetical protein from *B. uniformis* (PDB ID: 4KQ7) is available on the PDB from a structural genomics project, no functional information about DUF2961 is available. Importantly, αFFase1 does not show significant amino acid sequence similarity to any known enzymes, not just GHs. According to the results of this study, the Carbohydrate-Active enZymes team will create a new GH family, GH172 (B. Henrissat and N. Terrapon, personal communication).

### Possible metabolic roles of αFFase1

αFFase1 exhibited both α-D-fructofuranosidase and α-D-arabinofuranosidase activities. Arabinofuranosyl units in plant glycans are exclusively L-isomers, and α-D-arabinofuranosides are rarely found. For example, the cell wall polysaccharides of mycobacteria (45) and corynebacteria (46), pilins of *Pseudomonas aeruginosa* (47), and lipopolysaccharide *O*-antigens of *Stenotrophomonas maltophilia* (48) were reported to contain α-D-arabinofuranoside. Considering that *B. dentium* is frequently isolated from human oral plaque and caries (49) and from feces of elderly individuals (28), we speculate that the natural substrates of αFFase1 are α-D-fructofuranosides (DFAs and DHLs) or difructose compounds (inulobiose and Fru*p*β2,1Fru) present in the human diet. The main products of the caramelized D-fructose promoted by acid ion-exchange resins are DHL II and DFA I (50). *B. dentium* strains isolated from dental caries and plaque did not ferment inulin (49), and five representative *Bifidobacterium* species (*Bifidobacterium adolescentis* JCM 1275, *Bifidobacterium longum* JCM 1217, *Bifidobacterium breve* JCM 1192, *Bifidobacterium pseudocatenulatum* JCM 1200, and *Bifidobacterium catenulatum* JCM 1194) did not use DFA III *in vitro* (51). However, our preliminary study revealed that *B. dentium* JCM 1195 can grow on DFA I as the sole carbon source (K. Fujita *et al*., unpublished results). BBDE_2040 (αFFase1) and BBDE_2039 (GH32) are highly likely intracellular enzymes because they do not have an N-terminal secretion signal sequence. The neighboring ABC transporter genes (*BBDE_2041-2043*) (Fig. 1*A*) seem to be responsible for importing substrates for αFFase1 into the *B. dentium* cell. BBDE_2039 or other GH32 β-D-fructofuranosidases in the genome (*e.g.*, BBDE_1523) may cleave inulobiose into two D-fructose molecules.

### Presence of αFFase1 homologs in microbial genomes

Phylogenetic analysis of αFFase1 homologs was performed. Manual selection of homologs in currently available microbial genomes, in which the catalytic pocket residues are conserved, produced 120 sequences with sequence identity >47.45%, sequence coverage >79%, and e-value < 5.0 × 10^−128^ (Figs. S15 and S16). BACUNI_00161, which is the molecular replacement template, was not included in this analysis because of its low amino acid sequence identity. The C-terminal helix of BACUNI_00161 connects two trimers to form a hexamer (Fig. S17*A*), whose oligomerization architecture is clearly different from that of αFFase1 (Fig. 5*A*). Putative −1 subsite residues in BACUNI_00161, including the two catalytic glutamate residues, are conserved with those of αFFase1 (Fig. S17, *A* and *B*). However, Cys304 appears to clash with the O2 atom of Fru*f* in the −1 subsite, and residues in the putative +1 subsite are not conserved, suggesting that the *Bacteroides* protein is not a DFA I synthase/hydrolase.

The phylogenetic tree in Figure S15 was mainly composed of bacteria in different classes isolated from soil, plant, and gut of mammals. This is consistent with the potential implication of αFFase1 homologs in the metabolism of fructose polymers in vegetables. Sixteen proteins from the fungal genomes constitute a relatively distant clade. AFUA_5G00510 from *A. fumigatus* Af293 (XP_748288.1), which shares 48.6% sequence identity with BBDE_2040, is included in this fungal clade, suggesting that it corresponds to the *A. fumigatus* difructose-anhydride synthase studied in the 1980s (21, 22). We noticed that αFFase1 homologs are highly conserved among commensal gut bacteria in bifidobacteria and the Clostridia class (*Dorea*, *Clostridium*, *Faecalimonas*, *Coprococcus*, Eubacteriales, and *Ruminococcus*). Moreover, the gene pairs *BBDE_2040* and *BBDE_2039* are present in the genomes of 6 of 10 strains of *B. breve* isolated from the feces of infants, adults, and elderly individuals (Table S6) (52). These facts imply that an unknown metabolic pathway involving DFA I synthase/hydrolase is present in the human gut bacteria to utilize caramelized sugars.

### Metabolic pathway of DFAs in microorganisms

Microbial metabolism of cyclic glucooligosaccharides (*e.g.*, cyclodextrin, cyclic α-maltosyl-(1→6)-maltose, and cycloalternan) is often part of a monopolizing metabolic pathway that is advantageous in the competition for starch acquisition by transiently changing the polysaccharide into special cyclic forms (53, 54, 55, 56, 57). Such metabolic pathways generally consist of three stages: synthesis of the cyclic oligosaccharides outside the cells by an extracellular enzyme, uptake across the cellular membrane by a transporter, and degradation by intracellular enzymes. The three-stage metabolic pathways of inulin *via* DFA III by extracellular DFA III-forming inulin fructotransferase and intracellular DFA III hydrolase were also suggested to be present in *Arthrobacter ureafaciens* and *Arthrobacter* sp. H65-7 (13, 17, 58, 59, 60, 61). As to DFA I, *Streptomyces davawensis* JCM 4913 has an extracellular GH91 DFA I-producing inulin fructotransferase (BN159_0187) (62). The putative genes of intracellular DFA I synthase/hydrolase (B159_0193; 51.4% identity with αFFase1) and GH32 β-D-fructofuranosidase (BN159_0194) are present in the genome, suggesting that *S. davawensis* uses inulin *via* a three-stage metabolic pathway *via* DFA I. However, in the *B. dentium* JCM 1195 genome, there are no putative GH91 inulin fructotransferase genes. The metabolic system for α-D-fructofuranoside-containing sugars in oral and intestinal bacteria may have emerged after humans began using fire to cook food. The metabolic system may be relevant to the opportunistic pathogenicity of *B. dentium* in the development of dental caries (63). We will continue to study the metabolic pathway of *B. dentium* that involves αFFase1.

## Experimental procedures

### Materials and chemicals

Standard samples of DFA I and α-D-Fru*f*-(2→6)-D-Glc were kindly provided by Ohtaka Enzyme Co Ltd (26, 64). DFA III was purchased from FUJIFILM Wako Chemicals. Inulobiose and DFA I were prepared as follows: Inulobiose was prepared from DFA III by the enzymatic treatment of DFA III hydrolase from *Arthrobacter* sp. H65-7, as previously described (60). DFA I was also prepared from DFA III by cotreatment with DFA III hydrolase and αFFase1. The remaining inulobiose and DFA III were removed by cultivation with *Arthrobacter* sp. H65-7 cells. Inulobiose and DFA I were purified using a combination of an Autoprep FiberAC activated carbon column (Showa Denko) and HPLC on a COSMOSIL PBr column (φ 4.6 × 250 mm; Nacalai Tesque Inc). D-Fru*f*-α-Me and D-Ara*f*-α-Me were synthesized by methanolysis, as described by Bethell and Ferrier (65) and Sanki *et al*. (66), respectively. The synthesized sugar derivatives were purified using the aforementioned activated carbon column and COSMOSIL PBr column.

*p*NP-α-D-Ara*f* was synthesized, as previously described for the synthesis of arabinofuranoside derivatives (67) and enantiomeric L-Ara*f* derivatives (68, 69, 70). D-Arabinose was treated with tert-butyl(chloro)diphenylsilane in pyridine at 0 °C to afford 5-*O*-tert-butyldiphenylsilane (TBDPS) protected D-arabinofuranose in the furanoside form. This was followed by a one-pot treatment with acetic anhydride at room temperature overnight to obtain the corresponding 1,2,3-tri-*O*-acetyl-5-*O*-TBDPS protected D-arabinofuranose. After the introduction of thioglycoside at the anomeric position with 4-methylbenzenethiol in the presence of boron trifluoride etherate in dichloromethane at 0 °C, the TBDPS group was converted to acetate with tetra-*n*-butylammonium fluoride in tetrahydrofuran followed by acetic anhydride in pyridine (67). Glycosylation of the resulting thioglycoside with *para*-nitrophenol in NIS–silver(I) triflate in dichloromethane with MS 3A at 0 °C for 1 h afforded α-arabinofuranoside stereoselectively through neighboring group participation. Finally, conventional deprotection with NaOMe in methanol (MeOH) at 0 °C for 15 min gave *p*NP-α-D-Ara*f*. The structure of the product was verified by ^1^H NMR in D_2_O as the α-isomer (6.01 ppm, doublet, ^3^*J*_H1–H2_ = 1.2 Hz, C1-H) (71).

*p*NP-α-D-Fru*f* was synthesized using the method for the synthesis of *p*NP-α-D-Ara*f*. The furanoside formation of D-fructose with tert-butyl(chloro)diphenylsilane in pyridine followed by a one-pot treatment with acetic anhydride afforded Fru*f* protected by 2,3,4-tri-*O*-acetyl-1,6-di-*O*-TBDPS. After the introduction of the thioglycoside two-step conversion of 1,6-di-*O*-TBDPS to the corresponding acetate, selective glycosylation with *para*-nitrophenol followed by conventional deprotection afforded α-D-Fru*f*. Finally, conventional deprotection with NaOMe in MeOH at 0 °C for 15 min gave *p*NP-α-D-Fru*f* after separation by preparative TLC (eluent:CHCl_3_–MeOH = 10:1) on silica gel. The structure of the product was verified as the α-isomer from the peak at 113.1 ppm for C2 in the ^13^C NMR spectra and the peak at 4.21 ppm (doublet, ^3^*J*_H3–H4_ = 3.6 Hz) for C3-H in the ^1^H NMR spectra because C2 of α-Fru*f* generally appears in the lower field, and the coupling constant ^3^*J*_H3–H4_ of α-one is generally a smaller value than that of β-one, respectively (72). NMR data of *p*NP-α-D-Ara*f* are shown in supporting information. *p*NP-α-D-Fru*f* was found to be unstable under the conditions at 37 °C, even without the enzyme, possibly because of the nucleophilicity of the hydroxymethyl group at the anomeric carbon, which is not present in the *p*NP-α-D-Ara*f* molecule. Therefore, we used *p*NP-α-D-Ara*f* as a suitable substrate because it is stable and easy to be used for analyzing all furanose and pyranose anomers based on the peaks of C1-H with the values of ^3^*J*_H1–H2_ in the monitoring of the reaction by NMR.

### Production and purification of recombinant protein

The *B. dentium* JCM 1195 genome was extracted using a FastPure DNA kit (TaKaRa Bio). The primer sets indicated in Table S7 were used to amplify a DNA fragment corresponding to αFFase1 (locus tag BBDE_2040) using PrimeSTAR HS DNA Polymerase (TaKaRa Bio). A linearized pET23d(+) plasmid vector was prepared in the same manner using the corresponding primer set. The two DNA fragments were conjugated using an In-Fusion HD cloning kit (TaKaRa Bio). *E. coli* BL21 (DE3) (Agilent Technologies) was transformed with the αFFase1 expression vector and cultured in the LB medium containing 100 μg/ml ampicillin at 37 °C until the absorbance at 600 nm reached 0.4 to 0.6. The gene expression was induced by the addition of IPTG at a final concentration of 0.1 mM, and the culture proceeded for another 20 h at 25 °C. The bacteria were recovered by centrifugation and suspended in a lysis buffer composed of 50 mM Tris HCl (pH 7.0) and 300 mM NaCl. The suspension was sonicated using a model 250D sonifier (Branson Ultrasonics Corporation). The lysate was centrifuged, and the supernatant was applied to a column filled with cOmplete His-tag Purification Resin (Roche Diagnostics GmbH). The column had been preequilibrated with the lysis buffer. The column was washed with the lysis buffer supplemented with 5 mM imidazole, and the protein was eluted with the lysis buffer supplemented with 500 mM imidazole. The eluate was collected and concentrated using Amicon Ultracel-10K centrifugal filters (Millipore). The sample was loaded onto a Hiload 16/60 Superdex 200 pg column (Cytiva), preequilibrated with 25 mM Tris HCl (pH 7.0) and 300 mM NaCl. Approximately 50 to 70 mg of the target protein per liter culture with a purity of >99% was typically obtained after the sequential column-purification steps. Fractions corresponding to the target protein were collected and concentrated, and the buffer was exchanged with 5 mM sodium acetate (pH 6.0) using Amicon Ultracel-10K centrifugal filters. The purified protein was stored at 4 °C at a concentration higher than 10 mg/ml until further use. Protein sample purity was verified by SDS-PAGE during purification and conservation and analyzed by GelAnalyzer v.19.1. The protein concentration was determined by the bicinchoninic acid assay using bovine serum albumin (TaKaRa Bio) as the reference standard protein. For crystallization sample, which required a higher concentration, the protein concentration was determined by measuring the absorbance at 280 nm using a Nanodrop 2000 spectrophotometer (Thermo Fisher Scientific) using the molar extinction coefficient of 107,175 M^−1^ cm^−1^ that was calculated from the amino acid sequence.

### Enzyme assay and characterization

For every type of the substrate, enzyme activity was investigated by TLC and HPAEC-PAD. For TLC, a developing solvent of 1-butanol:2-propanol:water (10:5:4) was used, and orcinol sulfate was used as a visualizing solution. HPAEC-PAD was performed using the CarboPac PA1 column (Thermo Fisher Scientific). Elution occurred at a flow rate of 1.0 ml/min using the following gradient: 0 to 5 min, 100% eluent A (0.1 M NaOH); 5 to 30 min, 0 to 100% eluent B (0.5 M sodium acetate and 0.1 M NaOH); and 30 to 35 min, 100% eluent B. Carbohydrate concentrations were adjusted for the peak areas of standard samples. Calibration curves for DFA I and inulobiose up to 10 μM (Fig. S18) and 2 μM were measured, and quadratic equations were used for calculating concentration of the compounds. To determine the kinetic parameters of αFFase1 toward inulobiose and DFA I, 5 μg/ml (94.37 nM) of the enzyme was mixed with its substrate at different concentrations in 50 mM sodium acetate (pH 6.0) at 37 °C, and the reaction mixture was subjected to HPAEC-PAD over time. The *S*-*v* plot was analyzed by linear curve regression using SigmaPlot 12.0.

Inulobiose was also used to determine the effect of metal ions on the ligand and enzyme activity. αFFase1 was incubated for 24 h at 4 °C with the solution described above that was depleted of inulobiose and supplemented with 1 mM of divalent ions. Inulobiose was added, and the reaction occurred at 37 °C.

For *p*NP-α-D-Ara*f*, the activity was monitored by stopping the reaction with 4 volumes of 1 M sodium carbonate. The amount of released *p*NP was spectrophotometrically determined by measuring the absorbance at 400 nm. The reaction solution was composed of 0.1 μg/ml (1.89 nM) αFFase1, 100 mM sodium acetate (pH 6.0), and different concentrations of *p*NP-α-D-Ara*f* at 37 °C. The *S*-*v* plot was fit to the Michaelis–Menten equation using the enzyme kinetics tool from SigmaPlot 12.0. *p*NP-α-D-Ara*f* was also used to determine the optimal pH and temperature of αFFase1.

### Purification of Frupβ2,1Fru and DHL II from caramelized sugar

Caramelized sugar was prepared, as previously described (64) by heating D-fructose (60 g) at 130 °C for 120 min. For the mixture of fructose and glucose, 30 g of D-fructose and 30 g of D-glucose were used. The obtained solution was diluted in distilled water to a final concentration of 300 g/l. The remaining D-fructose was consumed by a baker's yeast fermentation process. Fru*p*β2,1Fru and DHL II were separated from fructose caramel by gel-filtration chromatography on a Bio-Gel P-2 column (φ 25 × 830 mm; Bio-Rad Laboratories) equilibrated with 1% sodium acetate. Subsequently, the oligosaccharide was purified using a combination of an Autoprep FiberAC activated carbon column (Showa Denko) and a COSMOSIL PBr column (φ 4.6 × 250 mm; Nacalai Tesque Inc) preequilibrated with 20 mM sodium phosphate (pH 2.5). Finally, Fru*p*β2,1Fru and DHL II were purified using a COSMOSIL PBr column with water as the mobile phase.

### NMR and MS analyses

#### Assignment of the substrate and the product

The collected fractions (peak 1 purified from caramelized sugar and peak 3 after treatment with αFFase1) were concentrated by freeze-drying, and the samples in D_2_O were analyzed by NMR (Fig. S6 for peak 3 and Fig. S8 for peak 1). These fractions were acetylated with acetic anhydride in pyridine. Acetylated peak 3 was analyzed by NMR in CDCl_3_ for further verification (Fig. S7). MALDI-TOF mass analysis with 2-hydroxy-5-methoxybenzoic acid as the matrix of peak 1 and 3 samples was performed. The results suggested that the product should be an anhydride form (*m*/*z* = 346.5 Da) of the disaccharide (*m*/*z* = 364.5 Da). MALDI-TOF mass analysis of both nonacetylated and acetylated fractions was also performed to estimate the number of hydroxy groups in the sample at peak 1 (*Δ*_acetylated – nonacetylated_ = 336 Da, eight acetyl groups) and peak 3 (*Δ*_acetylated – nonacetylated_ = 252 Da, six acetyl groups). The findings suggested that the anhydride was afforded from the disaccharide as the product (Fig. S5).

#### Verification of the stereochemistry of the initial product during hydrolysis of glycosidic linkage

The hydrolysis reaction was performed with 10 mM *p*NP-α-D-Ara*f* in D_2_O. A portion of the substrate (600 μl of a 10 mM solution in D_2_O) was mixed with 1 μl of the enzyme solution (30 mg/ml or 566 μΜ in water). The ^1^H NMR spectra of the reaction mixtures were recorded at 37 °C using an ECX400 spectrometer (JEOL) operating at 400 MHz (Fig. 2*A*). The ^1^H NMR spectra of the authentic samples, such as the initial *p*NP-α-D-Ara*f* and D-arabinose, which reached equilibrium, were obtained without enzyme addition. Using the spectra of the hydrolysis after 1 min, the ^1^H data of α-D-Ara*f* in the mixture were corrected and assigned using the relationship of the ^3^*J*_H–H_ coupling constants shown in Figure S3. The formation of α-D-Ara*f* in the reaction mixture was clearly observed in the anomeric region as the initial major furanoside (Fig. 2*B*).

#### Monitoring of the equilibrium between inulobiose and DFA I

Monitoring of the equilibrium between inulobiose and DFA I was performed using HPAEC-PAD (Figs. S11 and 4*B*) and NMR (Fig. S10*A*). A portion of inulobiose or enzymatically obtained DFA I (final concentration, 10 mM) was mixed with 50 μl of the enzyme solution in 50 mM sodium phosphate buffer (pH 6.0), with a total volume of 600 μl. The molar ratio of DFA I to inulobiose was monitored by the time course of the reaction of αFFase1 on HPAEC-PAD. The ^1^H NMR spectra of the reaction mixtures after 0 min (initial) and 24 h (after equilibrium) were recorded at 37 °C in the same manner as Figure 2*A*. The spectra are presented in Figure S10*A*. The data of the product (DFA I) shown in Figure S10*B* unambiguously indicated by ^1^*J*_H3–H4_ (2.4 Hz) and ^13^C chemical shifts of C3 at 82.1 ppm that the →3)-α-Fru*f* structure was formed from inulobiose.

### Protein crystallography and substrate modeling

Crystallization was performed by the sitting drop vapor diffusion method at 20 °C by mixing a protein solution (10 mg/ml or 189 μM αFFase1 in 5 mM Na acetate, pH 6.0) with a reservoir solution containing 20% (w/v) PEG 3350 and 0.2 M disodium tartrate at a 1:1 volume ratio. Cocrystals were prepared using a protein solution containing 80 mM D-arabinose or 20 mM D-fructose. The crystals were cryoprotected in a reservoir solution supplemented with 5%–10% 2-methyl-2,4-pentanediol and flash-cooled by dipping in liquid nitrogen.

Diffraction data were collected on beamlines at the Photon Factory of the High Energy Accelerator Research Organization (KEK) and Swiss Light Source (Paul Scherrer Institute). The diffraction datasets were processed using XDS (73) and Aimless (74). Molecular replacement was performed using MoRDa (75). Automated model building and refinement were performed using PHENIX (76). Manual model rebuilding was performed using Coot (77). Molecular graphics were prepared using PyMOL (Schrödinger, Inc).

A model of DFA I retrieved from MolView (https://molview.org) was placed in the active site of αFFase1 by superimposing the five-sugar ring atoms of one Fru*f* onto the β-Fru*f* molecule in the complex structure. Furanose conformations of the model were ^4^*T*_5_ (*p* = 30.6°) and *E*_3_ (*p* = 342.2°) for the sugars in −1 and +1 subsites, respectively. The model was energy-minimized using the sculpting wizard of PyMOL.

### Site-directed mutagenesis

Site-directed mutagenesis was performed using the complementary primer sets listed in Table S7. The mutant plasmid was amplified using KOD One PCR Master Mix (Toyobo). The PCR product was treated for 1 h with the DpnI restriction enzyme (TaKaRa Bio) at 37 °C. The linearized plasmid was transformed into *E. coli* JM109 (Agilent Technologies) for homologous recombination and amplification. The plasmid was then extracted, and the sequence was checked before being transformed into *E. coli* BL21 Star (DE3) (Invitrogen). Production and purification of the mutant proteins were performed in the same manner as the WT protein. Enzymatic activity was studied as described above, except that the substrate concentration was fixed at 2.5 mM for *p*NP-α-D-Ara*f* and 10 mM for inulobiose.

### MD simulations

MD simulations were performed for three forms: the αFFase1–β-d-Fru*f* complex, αFFase1–inulobiose complex, and αFFase1–DFA I complex. The initial structures of the MD simulations were prepared in the same manner, except that the crystal structure was used for the MD simulation of the αFFase1–β-d-Fru*f* complex, and the modeled structures were used for the MD simulations of the other two complexes. In each initial structure, the entire hexameric structure was used and the ligand was bound to all the protomers. The N and C termini of each protomer were capped with acetyl and *N*-methyl groups, respectively. The protonation states were determined based on the hydrogen bond network. His16, His207, and His262 were protonated at the Nδ1 atoms, and His 344 was protonated at both the Nδ1 and Nε2 atoms. The other histidine residues were protonated at the Nε2 atoms. After these modifications, the structure was immersed in a cubic box of water, ensuring a minimum distance of 10 Å between any box face and any protein atom. Potassium ions were added, so that the net charge of the system was zero. The dimensions of each system were 151 × 151 × 151 Å and comprised six identical protein chains, six identical ligands, 102 potassium ions, and approximately 88,000 water molecules. The Amber ff14SB force field parameters (78) were used for the proteins and ions. GLYCAM 06j (79) was used for the ligands, and the TIP3P model (80) was used for water. After energy minimization and equilibration, production MD runs were performed for 1 μs. During the MD simulations, the temperature was maintained at 300 K using the velocity-rescaling method (81) and the pressure was maintained at 1.0 × 10^5^ Pa using the Berendsen weak coupling method (82). The bond lengths involving hydrogen atoms were constrained using the LINCS algorithm (83, 84) to allow a time step of 2 fs. Electrostatic interactions were calculated using the particle mesh Ewald method (85, 86). All the MD simulations were performed using GROMACS 2020 (87), with coordinates recorded every 10 ps.

### Phylogenetic tree

The αFFase1 homolog sequences were retrieved from the BLAST alignment results using the reference protein database. Sequences with conserved active site residues were manually selected on MEGA and subjected to M-Coffee multiple sequence alignment. The alignment was used to produce a phylogenetic tree based on the maximum-likelihood algorithm, with a bootstrap of 1000 using MEGA. The final tree was displayed using iTOL v6 (88).

## Data availability

Atomic coordinates and structure factors (codes 7V1V, 7V1W, and 7V1X) have been deposited in the Protein Data Bank (http://wwpdb.org/).

## Supporting information

This article contains supporting information (52, 89).

## Conflict of interest

The authors declare that they have no conflicts of interest with the contents of this article.
