# Supplementary material for: Identification of difructose dianhydride I synthase/hydrolase from an oral bacterium establishes a novel glycoside hydrolase family
Source: J Biol Chem. 2021 Oct 22;297(5):101324. doi: 10.1016/j.jbc.2021.101324 (PMC8605356; doi:10.1016/j.jbc.2021.101324)
Supplement: Figures S1–S18 and Tables S1–S7 [file mmc3.pdf]

Supporting Information

Identification of a difructose dianhydride I synthase/hydrolase from oral bacterium establishes a novel glycoside hydrolase family

Toma Kashima (鹿島 騰真)[1‡], Kouki Okumura (奥村 公喜)[2‡], Akihiro Ishiwata (石渡 明弘)[3‡], Machika Kaieda (海江田 磨千加)[2], Tohru Terada (寺田 透)[1], Takatoshi Arakawa (荒川 孝俊)[1,4], Chihaya Yamada (山田 千早)[1,4], Kentaro Shimizu (清水 謙多郎)[1], Katsunori Tanaka (田中 克典)[3,5], Motomitsu Kitaoka (北岡 本光)[6], Yukishige Ito (伊藤 幸成)[3,7], Kiyotaka Fujita (藤田 清貴)[2,*], and Shinya Fushinobu (伏信 進矢)[1,4,*]

[1] Department of Biotechnology, The University of Tokyo, Tokyo, Japan

[2] Faculty of Agriculture, Kagoshima University, Kagoshima, Japan

[3] Cluster for Pioneering Research, RIKEN, Saitama, Japan

[4] Collaborative Research Institute for Innovative Microbiology, The University of Tokyo, Tokyo, Japan

[5] Department of Chemical Science and Engineering, Tokyo Institute of Technology, Japan

[6] Faculty of Agriculture, Niigata University, Niigata, Japan

[7] Graduate School of Science, Osaka University, Osaka, Japan

[‡]These authors contributed equally to this work.

[*]Correspondence to: Shinya Fushinobu, asfushi@mail.ecc.u-tokyo.ac.jp; Kiyotaka Fujita, k4022897@kadai.jp.

List of materials included

- **NMR data of 4-nitrophenyl α-D-fructofuranoside**
- **Figures S1-S18**
- **Legends for Supplementary Movie S1-S2**
- **Tables S1-S7**
- **References**

NMR data of 4-nitrophenyl α-D-fructofuranoside: ^{1}H NMR (400 MHz, CD_3OD): δ 3.60 (dd, *J* = 12.4, 4.0 Hz, C6-H, 1 H), 3.70–3.75 (m, C1-H, C6-H, 2 H), 3.79 (d, *J* = 12.4 Hz, C1-H, 1 H), 3.90–3.94 (m, C4-H, C5-H, 2 H), 4.21 (d, *J* = 3.6 Hz, C3-H, 1 H), 7.28–7.33 (m, Ar, 2 H), 8.06–8.10 (m, Ar, 2 H);^{13}C NMR (100 MHz, CD_3OD): δ 61.2 (C1), 62.7 (C6), 78.0 (C4), 83.9 (C3), 85.5 (C5), 113.1 (C2), 122.3 (*p*NP), 126.0 (*p*NP), 144.2 (*p*NP), 161.7 (*p*NP).

A

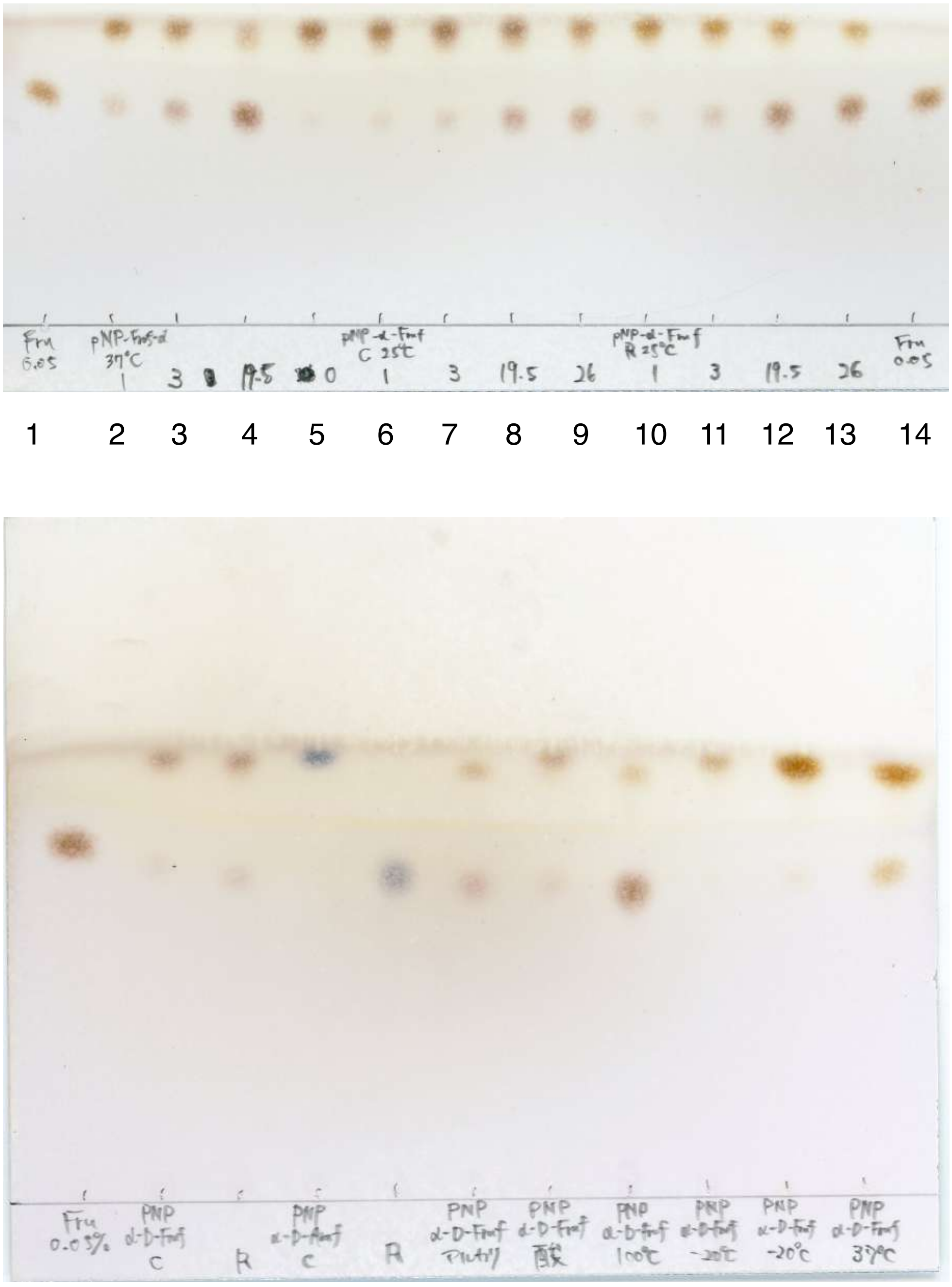

1 2 3 4 5 6 7 8 9 10 11 12 13 14

B

	1	2	3	4	5	6	7	8	9	10	11
*p*NP-D-Fru*f*		+	+	−	−	+	+	+	+	+	+
*p*NP-D-Ara*f*		−	−	+	+	−	−	−	−	−	−
αFFase1		−	+	−	+	−	−	−	−	−	−

Figure S1. TLC analysis of spontaneous degradation of *p*NP-α-D-Fru*f* and activity of αFFase1 (BBDE_2040) on *p*NP-α-D-Fru*f* and *p*NP-α-D-Ara*f*. *A*, Lanes 1 and 14, D-fructose. Lanes 2-13, *p*NP-α-D-Fru*f* (5.0 mM) was incubated in 25 mM sodium acetate buffer (pH 6.0) without αFFase1 at 37 °C for 1, 3, and 19.5 h (lanes 2-4), without αFFase at 25 °C for 0, 1, 3, 19.5, and 26 h (lanes 5-9),

and with 12.5 µg/mL αFFase1 at 25 °C for 1, 3, 19.5, and 26 h (lanes 10-13). *B*, Lane 1, D-fructose. Lanes 2-5, *p*NP-α-D-Fru*f* or *p*NP-α-D-Ara*f* (5.0 mM) was incubated in 25 mM sodium acetate buffer (pH 6.0) without or with FFase at 25 °C for 1 h. Lanes 6-7, *p*NP-α-D-Fru*f* (1.0 mM) was incubated without αFFase1 under alkaline condition incubated in 0.5 M sodium carbonate (lane 6) or acidic condition in 0.5 M sodium acetate (pH 4.0) (lane 7) at 25 °C for 1 h. Lanes 8, 10-11, *p*NP-α-D-Fru*f* (5.0 mM) was incubated without αFFase1 under varying temperatures in 25 mM sodium acetate buffer (pH 6.0) at 100 °C (lane 8), –20 °C (lane 10), and 37 °C (lane 11) for 3 h. Lane 9, *p*NP-α-D-Fru*f* (0.5 mM) was incubated without αFFase1 in 25 mM sodium acetate buffer (pH 6.0) at –20 °C for 3 h.

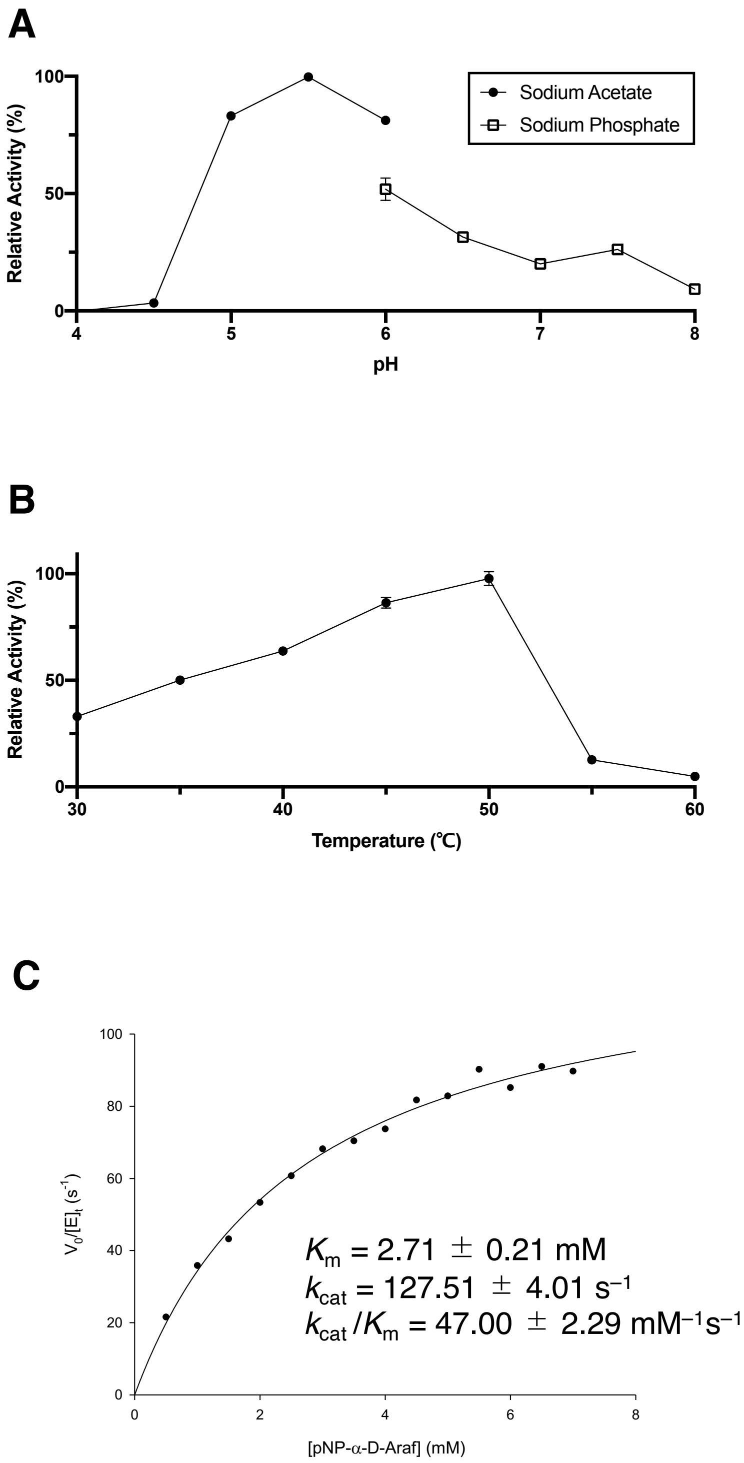

Figure S2. Enzymatic characterization of the activity of αFFase1 toward *p*NP-α-D-Ara*f*. Effects of pH at 37 °C (*A*), effects of temperature at pH 6.0 (*B*), and *S*-*v* plot at pH 6.0 and 37 °C (*C*) are shown.

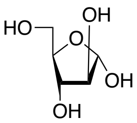

Chemical shift<br>δ (ppm)	Multiplicity and<br>Coupling constants (Hz)	Assignment
5.39	d 2.8	Araf$^{C1\text{-}H}$
4.27	ddd 6.4, 5.6, 3.6	Araf$^{C4\text{-}H}$
4.18	dd 4.8, 2.8	Araf$^{C2\text{-}H}$
4.13	dd 6.4, 4.8	Araf$^{C3\text{-}H}$
3.94	dd 12.4, 3.6	Araf$^{C5\text{-}H}$
3.83	dd 12.4, 5.6	Araf$^{C5\text{-}H}$

Figure S3. ^{1}H NMR data of initial isomer (α-D-Ara*f*) obtained from hydrolysis of *p*NP-α-D-Ara*f* with αFFase1. Data of a sample incubated with the enzyme for 1 min was collected at 400 MHz in D_2O at 37 °C.

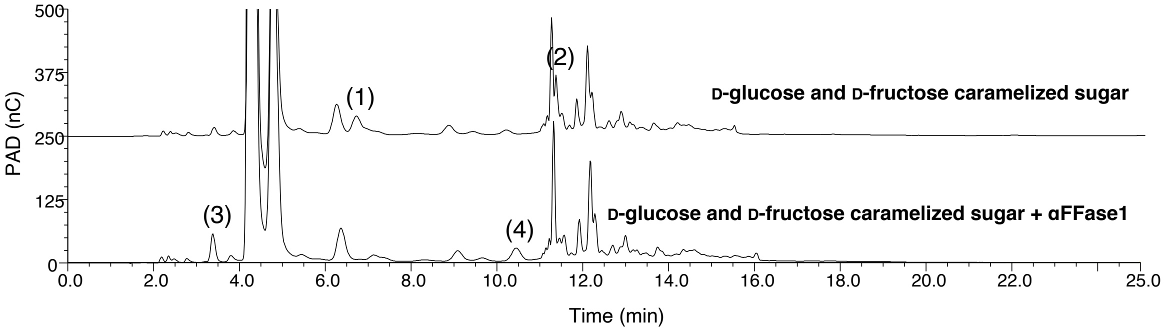

Figure S4. Activity of αFFase1 toward caramelized sugar made from D-fructose and D-glucose. HPAEC-PAD chromatogram of the caramelized sugar alone (top) and caramelized sugar treated with αFFase1 overnight at 37 °C (bottom). The same four peaks with those in Fig. 2*A* showed significant changes by the enzyme treatment from peaks 1 and 2 to peaks 3 and 4.

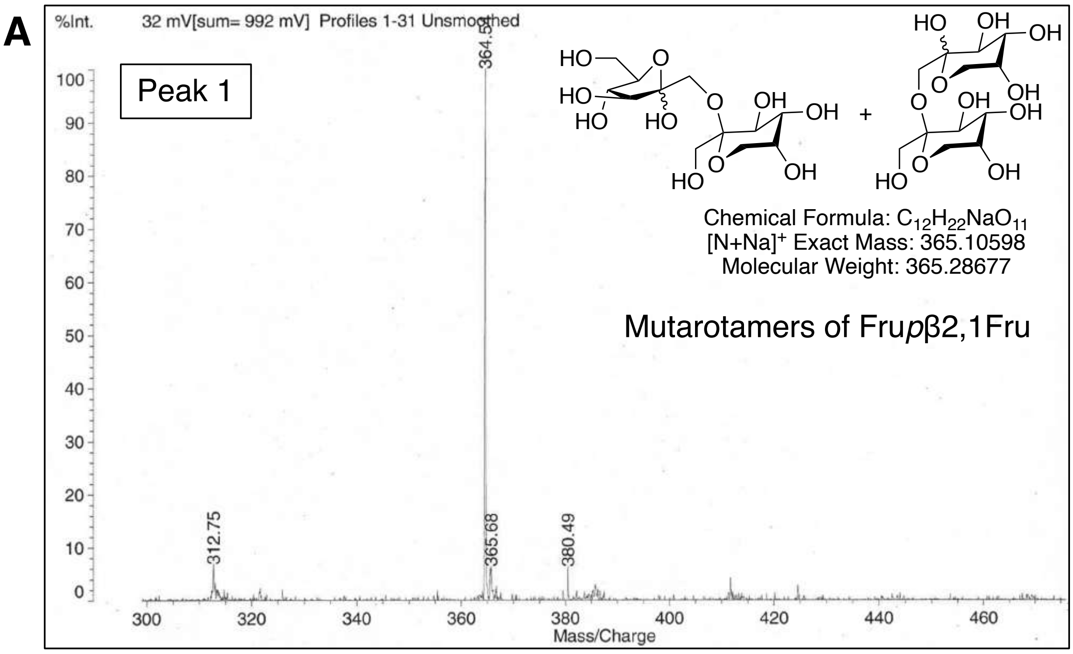

A
%Int.
32 mV[sum= 992 mV] Profiles 1-31 Unsmoothed
Peak 1
364.5
312.75
365.68
380.49
Mass/Charge
Chemical Formula: C12H22NaO11
[N+Na]+ Exact Mass: 365.10598
Molecular Weight: 365.28677
Mutarotamers of Frupβ2,1Fru

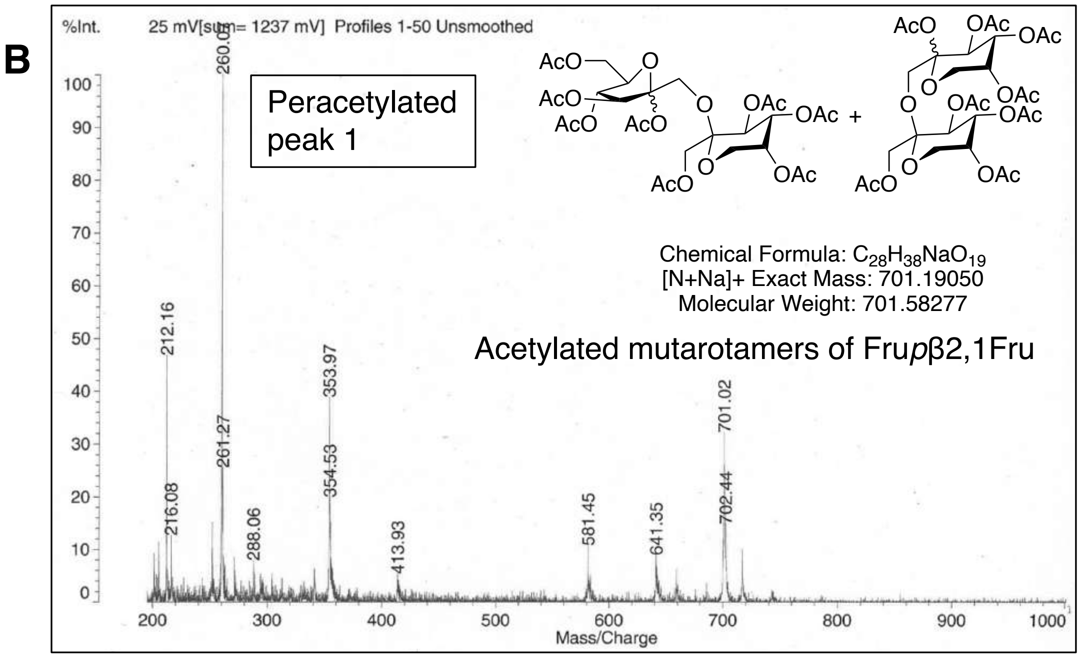

B
%Int.
25 mV[sum= 1237 mV] Profiles 1-50 Unsmoothed
Peracetylated peak 1
212.16
216.08
260.0
261.27
288.06
353.97
354.53
413.93
581.45
641.35
701.02
702.44
Mass/Charge
Chemical Formula: C28H38NaO19
[N+Na]+ Exact Mass: 701.19050
Molecular Weight: 701.58277
Acetylated mutarotamers of Frupβ2,1Fru

C

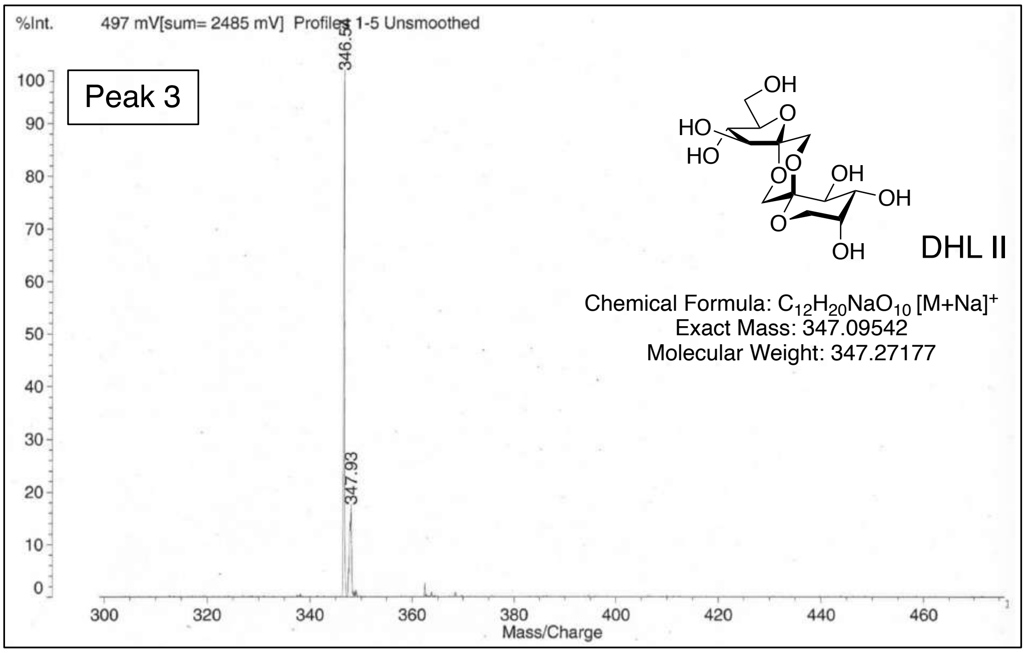

D

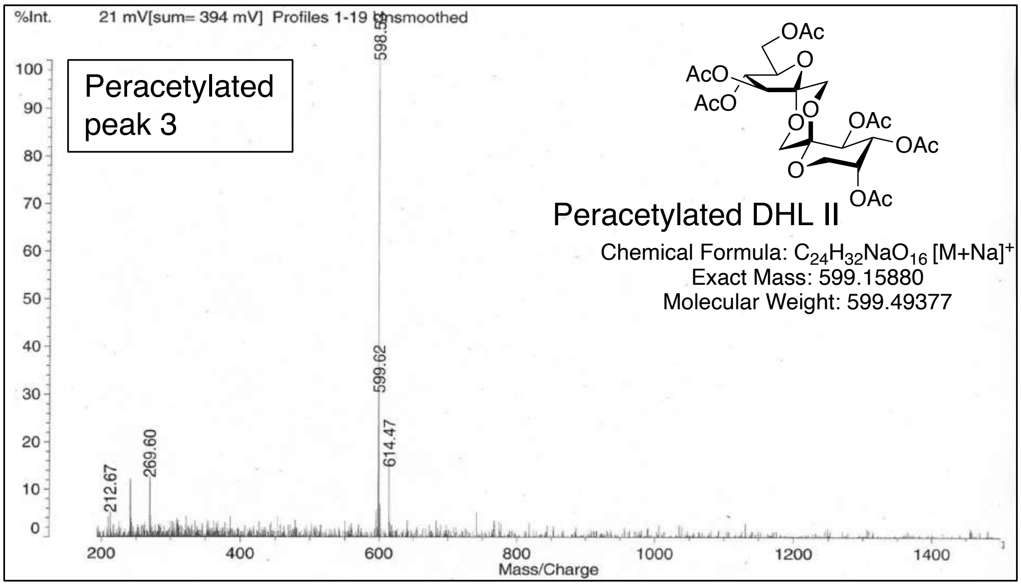

Figure S5. MALDI-TOF MS analysis of peaks 1 and 3, and their peracetylated samples. Mass spectra of peak 1 (*A*), peracetylated peak 1 (*B*), peak 3 (*C*), and peracetylated peak 3 (*D*) are shown. The observed ions were detected as Na^+ adducts. The increased difference of molecular weight (336 Da for peak 1 and *D* = 252 Da for peak 3) indicated 8 and 6 acetylations were occurred in each sample, respectively, since one acetyl substitution results in +42 Da.

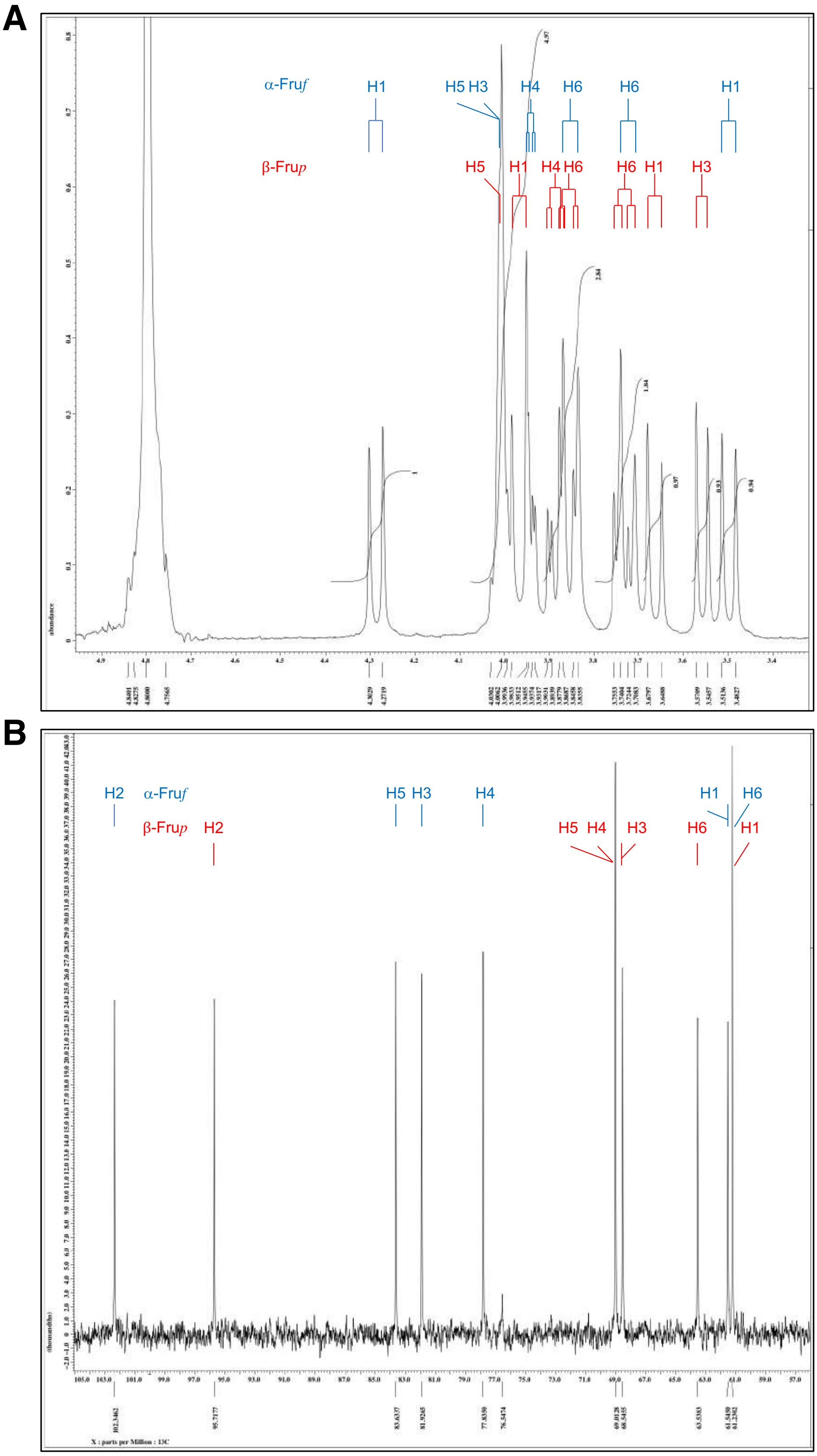
A
α-Fruf
H1
H5 H3
H4
H6
H6
H1
β-Frup
H5
H1
H4 H6
H6
H1
H3
B
H2
α-Fruf
H5 H3
H4
H1
H6
β-Frup
H2
H5 H4
H3
H6
H1
X : parts per Million : 13C

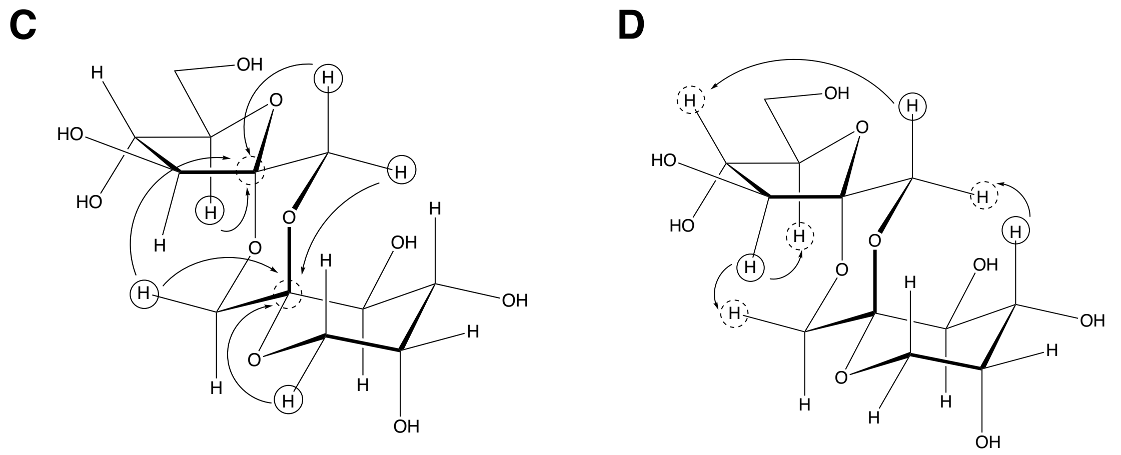

Figure S6. NMR spectra of the purified peak 3 (DHL II). *A*, ^{1}H-NMR spectrum (400 MHz in D_2O). *B*, ^{13}C-NMR spectrum (100 MHz in D_2O). *C*, Selected ^{1}H-^{13}C HMBC relationship indicated by arrows from protons (circle) to two C1 atoms (dashed circle) of the furanoside and pyranoside. *D*, Selected NOE relationship indicated by arrows from irradiated (circle) to enhanced (dashed circle) protons.

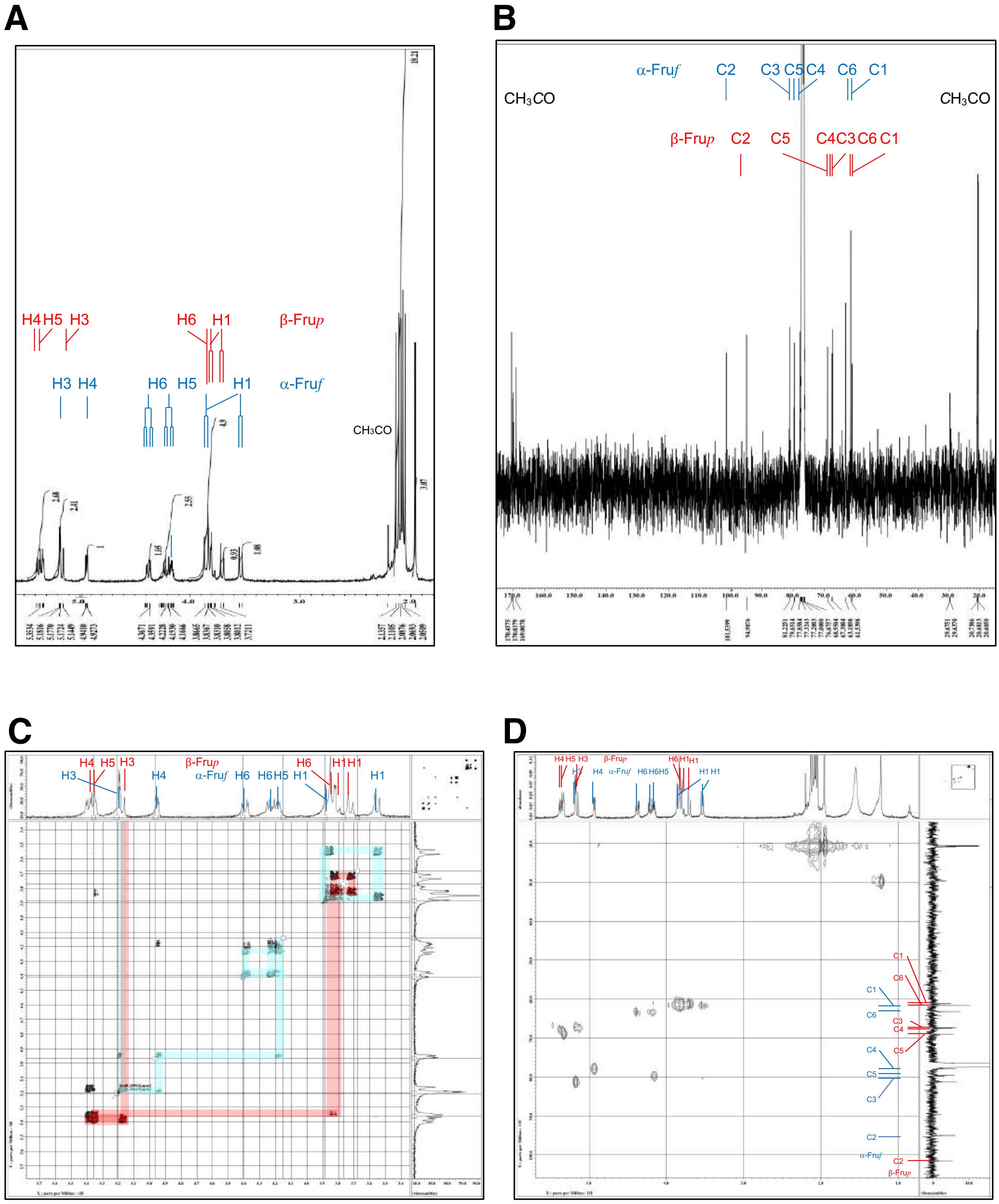
A
H4 H5 H3
H6 H1
β-Fru*p*
H3 H4
H6 H5 H1
α-Fru*f*
CH3CO
B
α-Fru*f*
C2
C3 C5 C4
C6 C1
CH3CO
β-Fru*p*
C2
C5
C4C3C6 C1
CH3CO
C
H4 H5 H3
β-Fru*p*
H6 H1 H1
H3
H4
α-Fru*f*
H6
H6 H5
H1
H1
D
H4 H5 H3
β-Fru*p*
H6 H1 H1
H3
H4
α-Fru*f*
H6 H6 H5
H1 H1
C1
C6
C1
C6
C3
C4
C4
C5
C5
C3
C2
α-Fru*f*
C2
β-Fru*p*

E

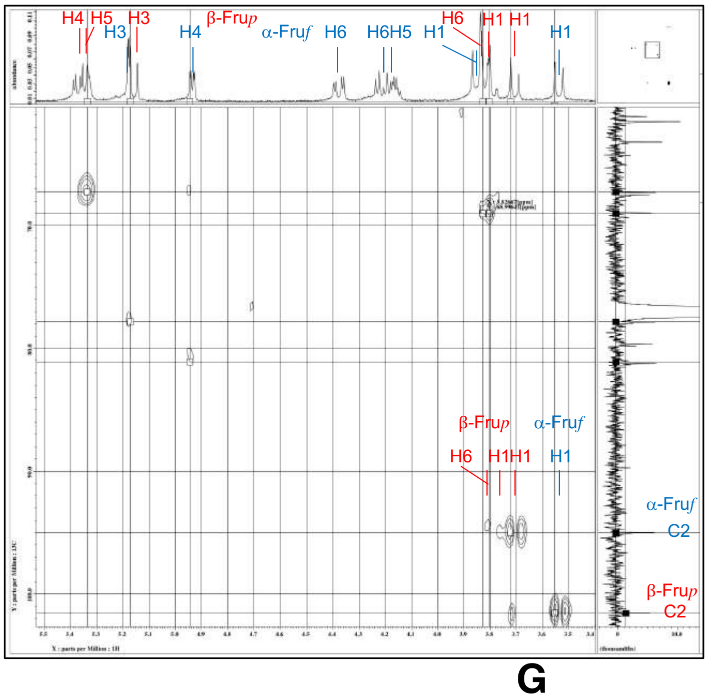

F

Frup	¹H	³J Hz	¹³C	Key HMBC
1	3.71	d, 12.0	61.12	→ 101.54 (Fruf--C2), 94.99 (Frup-C2)
	3.82	d, 12.0		
2	-	-	94.99	
3 (Ac)	5.16	d, 13.2	67.30	
4 (Ac)	5.37	dd, 10.0, 3.2	67.51	
5 (Ac)	5.33	m	68.95	
6	3.79	dd, 13.2 1.6	61.54	→ 94.99 (Frup-C2)
	3.84	d, 13.2,		

Fruf	¹H	³J Hz	¹³C	Key HMBC
1	3.55	d, 12.0	61.54	→ 101.54 (Fruf-C2)
	3.85	d, 12.0		
2	-	-	101.54	
3 (Ac)	5.18	d, 2.0	81.23	
4 (Ac)	4.94	dd, 5.6, 2.0	77.86	
5	4.16	td, 5.6, 3.2	79.65	
6 (Ac)	4.21	dd, 12.0, 3.2	63.19	
	4.38	dd, 12.0, 5.6		

	¹H	³J Hz	¹³C
Ac		s	170.46 (x2), 170.35, 170.04, 169.01(x2)
	2.14, 2.11, 2.09, 2.07, 2.05, 1.96	s	20.95, 20.74(x2), 20.68, 20.61(x2),

G

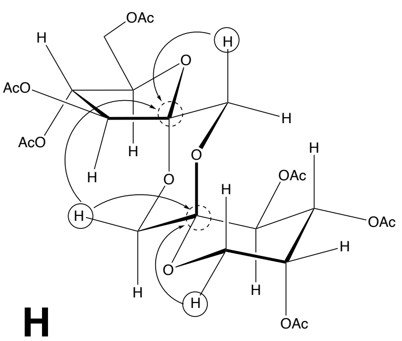

H

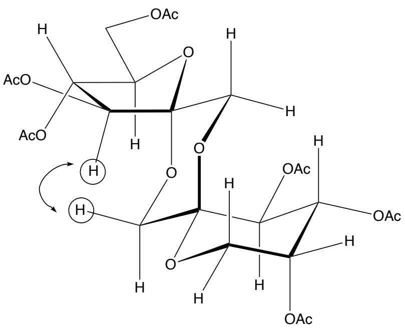

Figure S7. NMR analysis of the peracetylated peak 3 (DHL II) for detailed assignment. *A*, ^{1}H-NMR spectra (400 MHz in $CDCl_3$). *B*, ^{13}C-NMR spectrum (100 MHz in $CDCl_3$); C) ^{1}H-^{1}H COSY spectrum. *D*, ^{1}H-^{13}C HMQC spectrum. *E*, ^{1}H-^{13}C HMBC spectrum. *F*, List of NMR data of the peracetylated unknown product. *G*, Selected ^{1}H-^{13}C HMBC relationships indicated by arrows from protons (circle) to two C1 (dotted circle) of furanoside and pyranoside. *H*, Selected NOE relationship indicated by an arrow between irradiated (circle) protons.

A

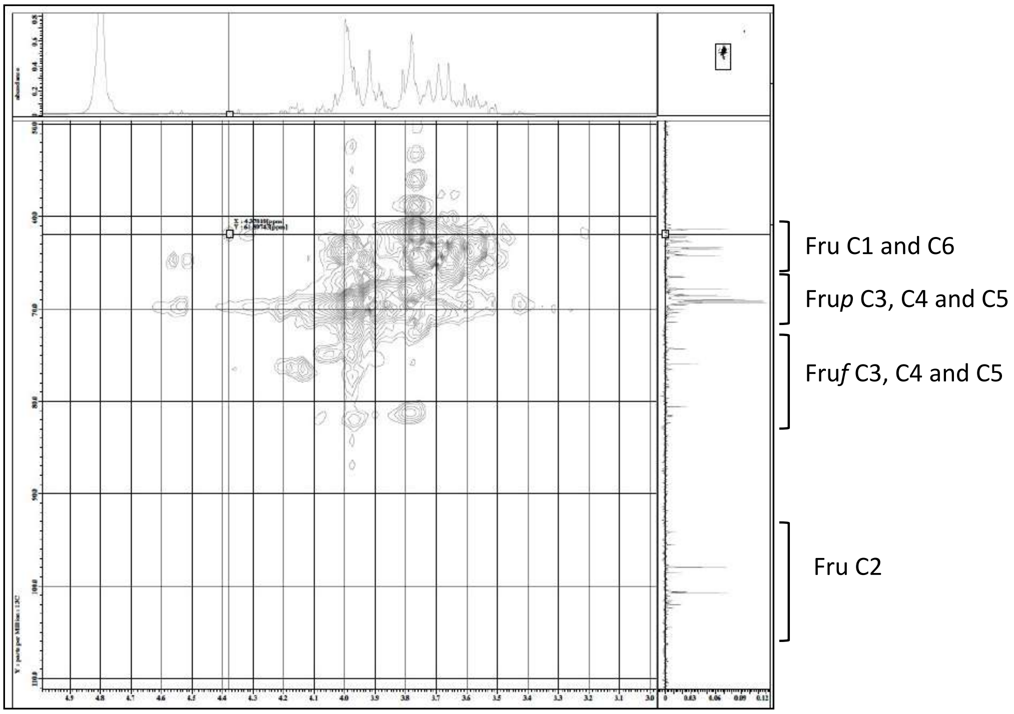

B

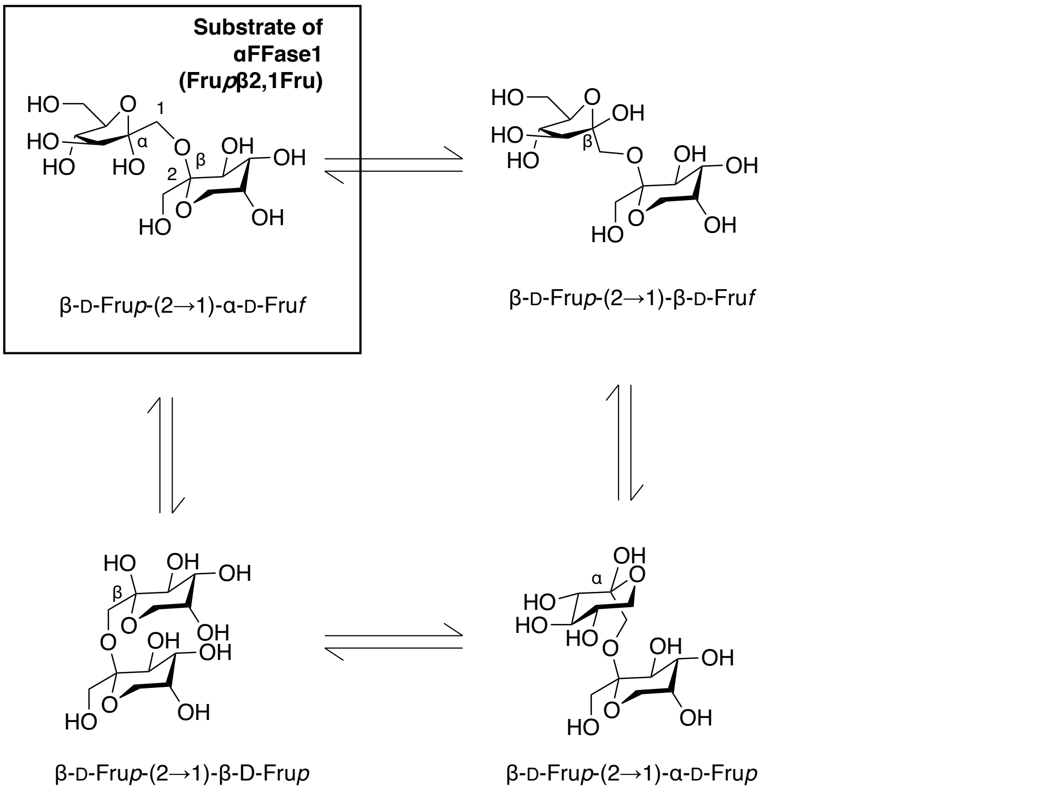

Figure S8. NMR analysis of the purified peak 1 (Fru*p*β2,1Fru). *A*, ^{1}H-^{13}C HMQC spectrum in D_2O. ^{13}C chemical shifts corresponding to certain atoms are indicated on the right. *B*, The predicted isomeric composition of peak 1 under mutarotation conditions. The expected substrate of αFFase1 (Fru*p*β2,1Fru containing α-D-fructofuranose) in the mutarotation mixture is boxed in a solid line.

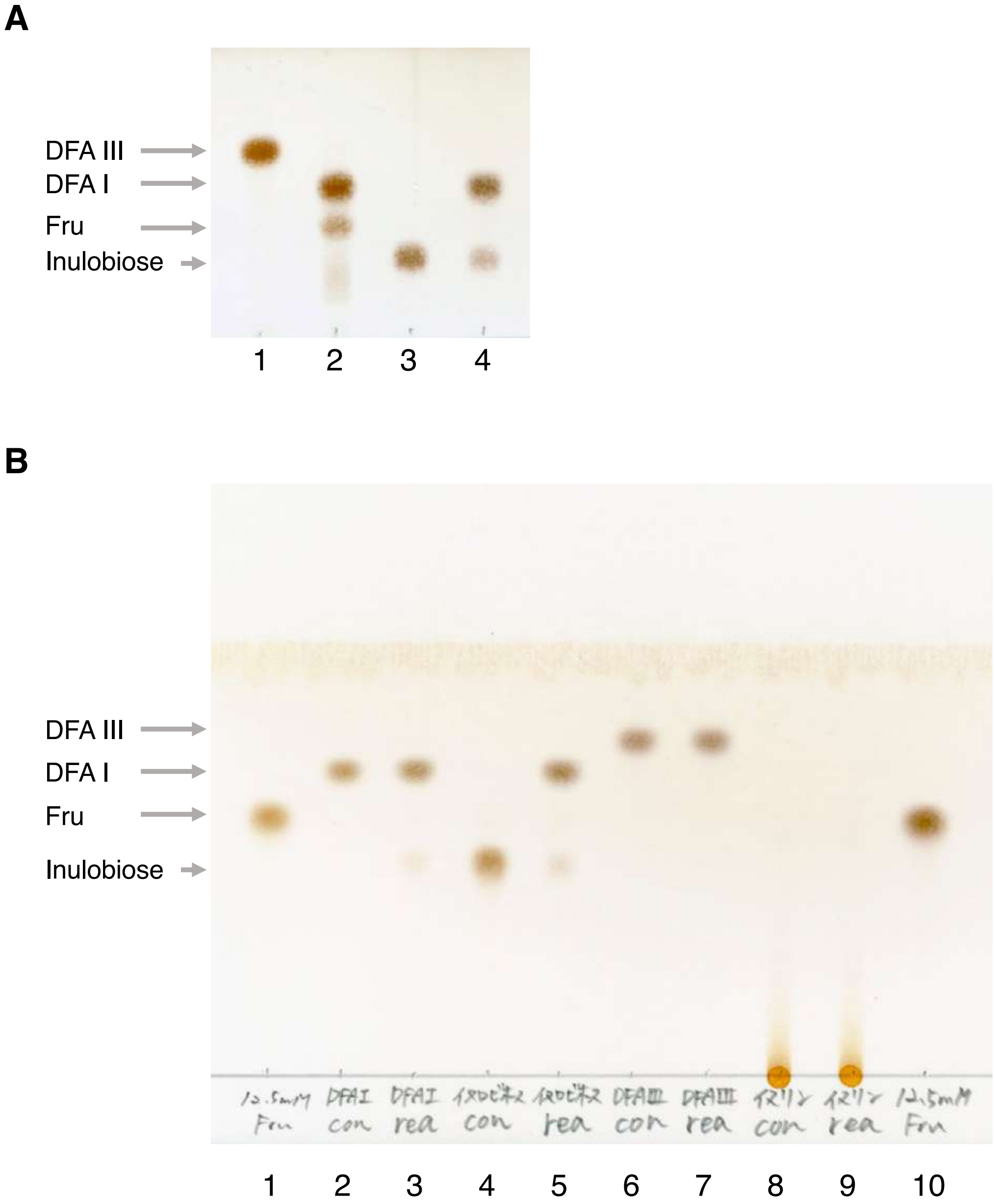

Figure S9. TLC analysis of reaction by αFFase1. *A*, Standard samples of DFA III (lane 1), DFA I (lane 2, 85% purity), and inulobiose (lane 3), and the reaction product of inulobiose with αFFase1 (lane 4). *B*, Standard samples of D-fructose (lanes 1 and 10), DFA I (lane 2), inulobiose (lane 4), DFA III (lane 6), and inulin (lane 7), and their reaction products with αFFase1 (lane 3, 2 mM DFA I; lane 5, 2 mM inulobiose; lane 7, 2 mM DFA III; and lane 9, 1% inulin). The reactions were performed in 50 mM Na-acetate (pH 6.0) overnight at 37 °C.

A

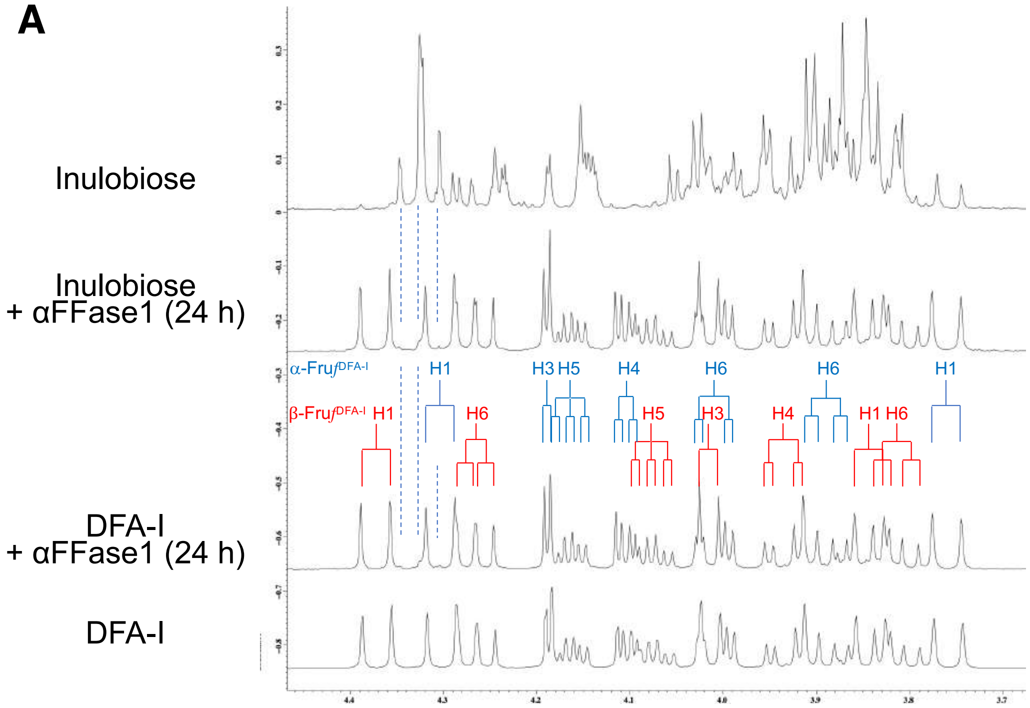

B

α-Fru*f*	¹H	³*J* Hz	¹³C
1	4.30	d 12.4	62.79
	3.76	d 12.4	
2	-	-	102.69
3	4.19	d 2.4	82.11
4	4.10	dd 6.0, 2.4	77.95
5	4.17	td 6.0, 3.2	83.68
6	4.01	dd 12.4, 3.2	61.42
	3.89	dd 12.4, 6.0	

β-Fru*f*	¹H	³*J* Hz	¹³C
1	4.37	d 12.4	61.98
	3.84	d 12.4	
2	-	-	99.08
3	4.01	d 8.4	77.22
4	4.27	dd 8.4, 7.2	74.84
5	4.08	td 7.2, 3.6	81.48
6	3.93	dd 12.4, 3.6	62.92
	3.81	dd 12.4, 7.2	

Figure S10. NMR monitoring of the reactions of αFFase1 toward inulobiose and DFA I. *A*, Inulobiose (top) or DFA I (bottom) was treated with αFFase1 to reach equilibrium (the second and third spectra). The reactions were monitored by 400 MHz ^{1}H-NMR in D_2O (10 mM sodium phosphate buffer, pH 6.0) at 37 °C for 24 h. *B*, ^{1}H and ^{13}C chemical shifts of DFA I that were enzymatically produced from inulobiose.

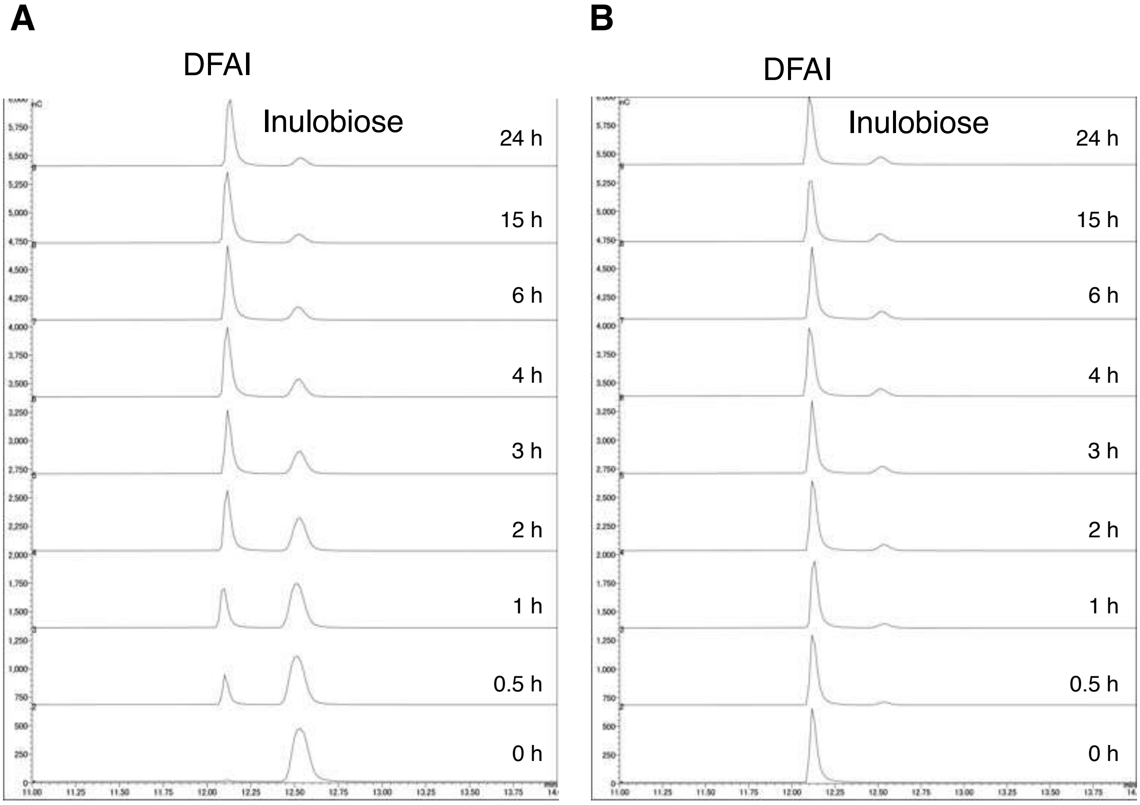

Figure S11. HPAEC-PAD chromatogram of αFFase1 reaction using inulobiose (*A*) or DFA I (B) as substrate. The time courses of the reactions are shown.

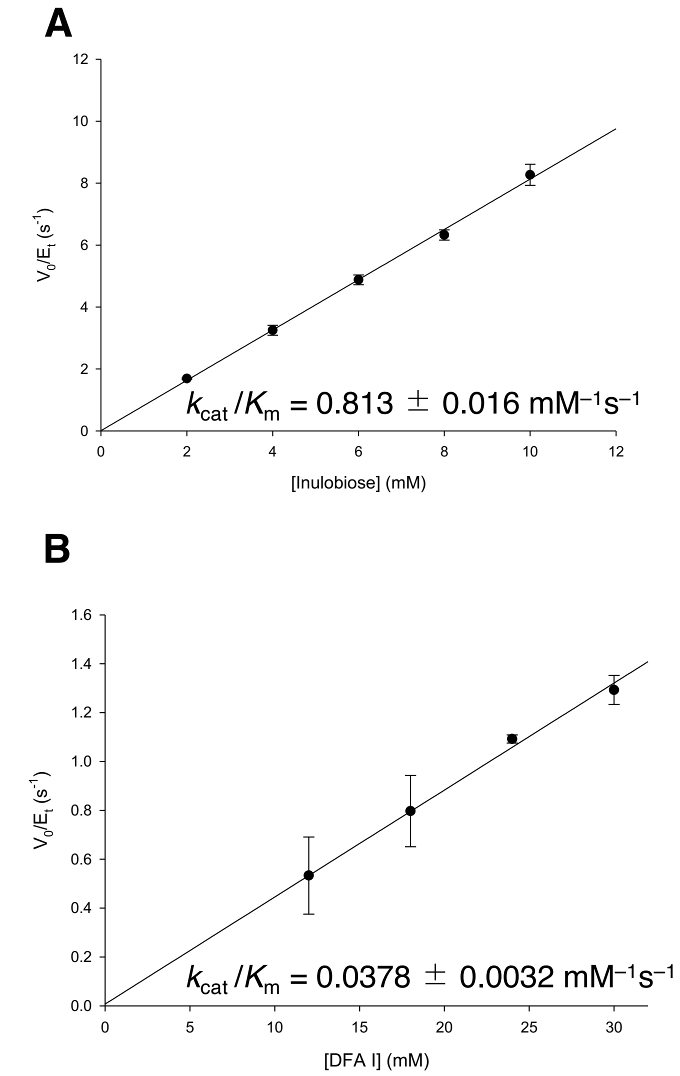

Figure S12. *S*-*v* plot of the αFFase1 reaction toward inulobiose (*A*) and DFA I (*B*) at pH 6.0 and 37 °C.

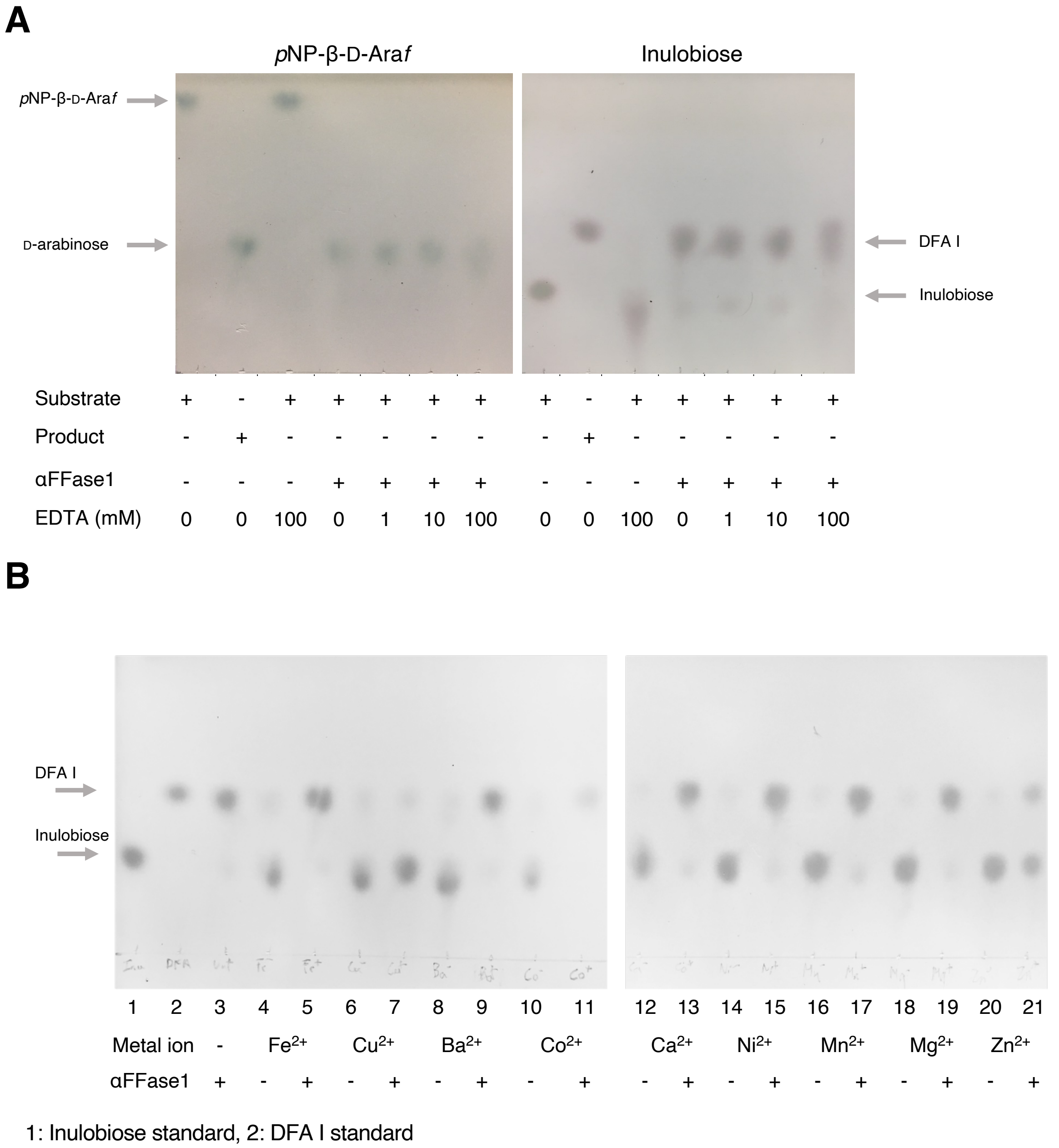

Figure S13. Effect of EDTA (*A*) and divalent metal ions (*B*) on the activity of αFFase1 toward *p*NP-α-D-Ara*f*. *A*, αFFase1 (20 µg/mL) was incubated in the presence of 0–100 mM EDTA in 100 mM Na-acetate (pH 6.0) overnight at 4 °C before the assay. The enzyme reaction was performed with αFFase1 (1 µg/mL) and 10 mM substrate (*p*NP-α-D-Ara*f* or inulobiose) in 100 mM Na-acetate (pH 6.0) and 0–100 mM EDTA at 37 °C for 1 h. D-Arabinose and DFA I were used as standards for the products. *B*, αFFase1 (20 µg/mL) was incubated in the presence of 0 or 1 mM divalent metal ion ($FeCl_2$, $CuCl_2$, $BaCl_2$, or $CoCl_2$) in 100 mM Na-acetate (pH 6.0) for 24 h at 4 °C before the assay. The enzyme reaction was performed with αFFase1 (1 µg/mL) in 100 mM Na-acetate (pH 6.0) and 0 or 1 mM divalent metal ion overnight at 37 °C.

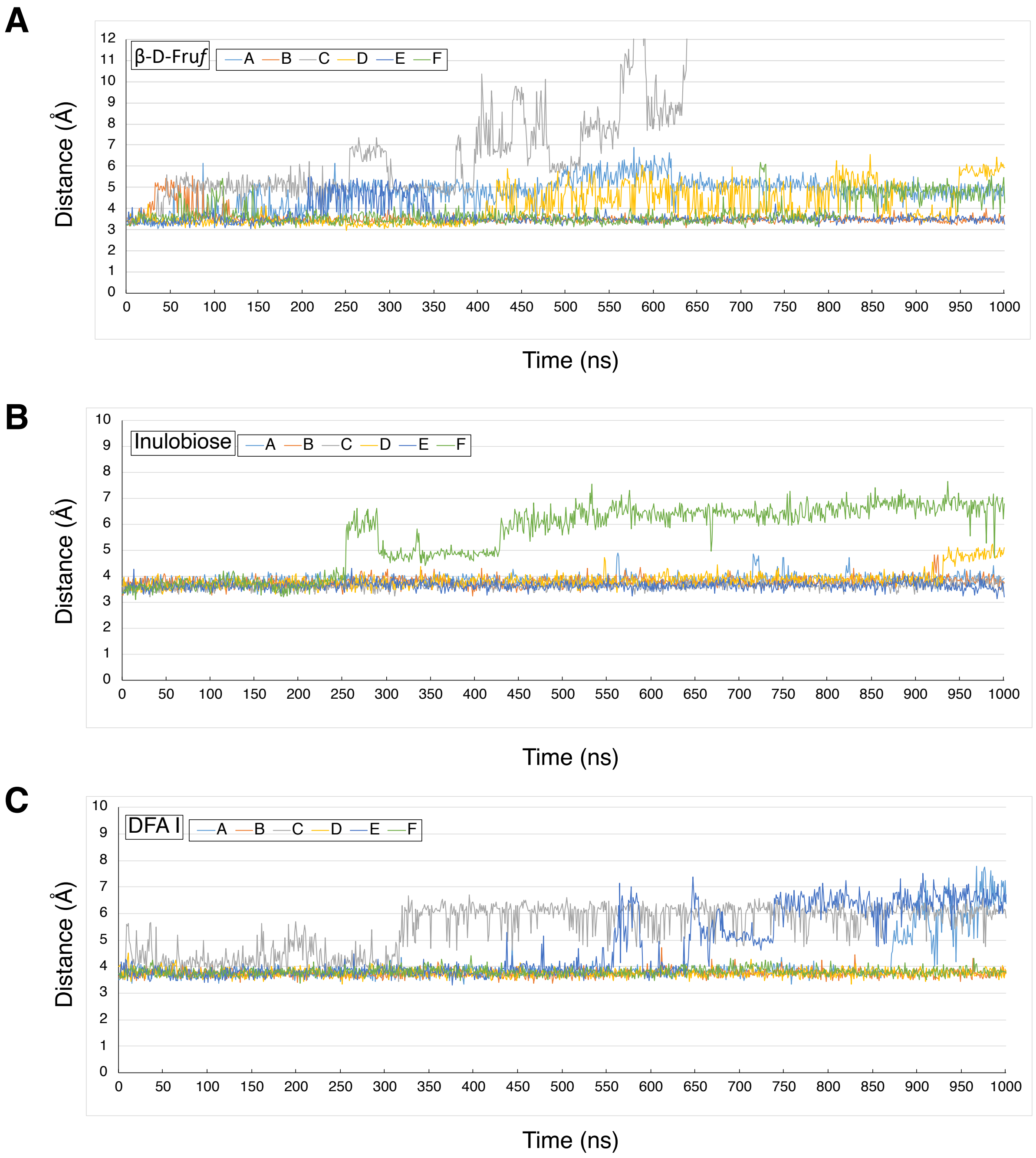

Figure S14. Stability of the ligand in the active site during MD simulation. Time evolution of the distance between the anomeric C2 atom of D-Fru*f* in −1 subsite and the nearest side chain oxygen atom of E291 (nucleophile) during the MD simulations of the complexes with β-D-Fru*f* (*A*), inulobiose (*B*), and DFA I (*C*) are plotted for each chain.

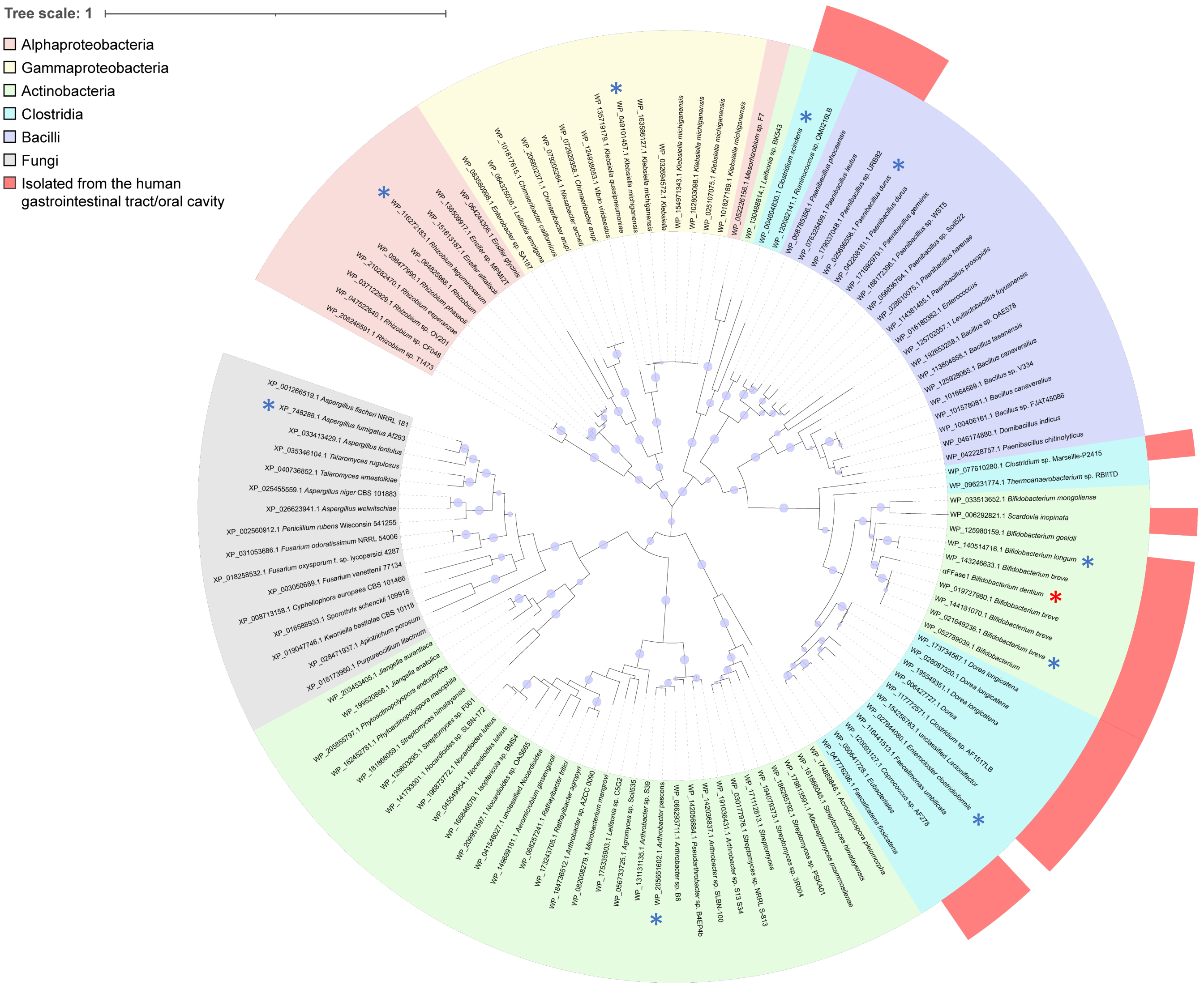

Figure S15. Phylogenetic tree of αFFase1 and its close homologs. Bootstrap robustness, ranging from 50 to 100% is shown with blue circles. αFFase1 is indicated with a red asterisk. Homologs shown in Fig. S16 are indicated with a blue asterisk.

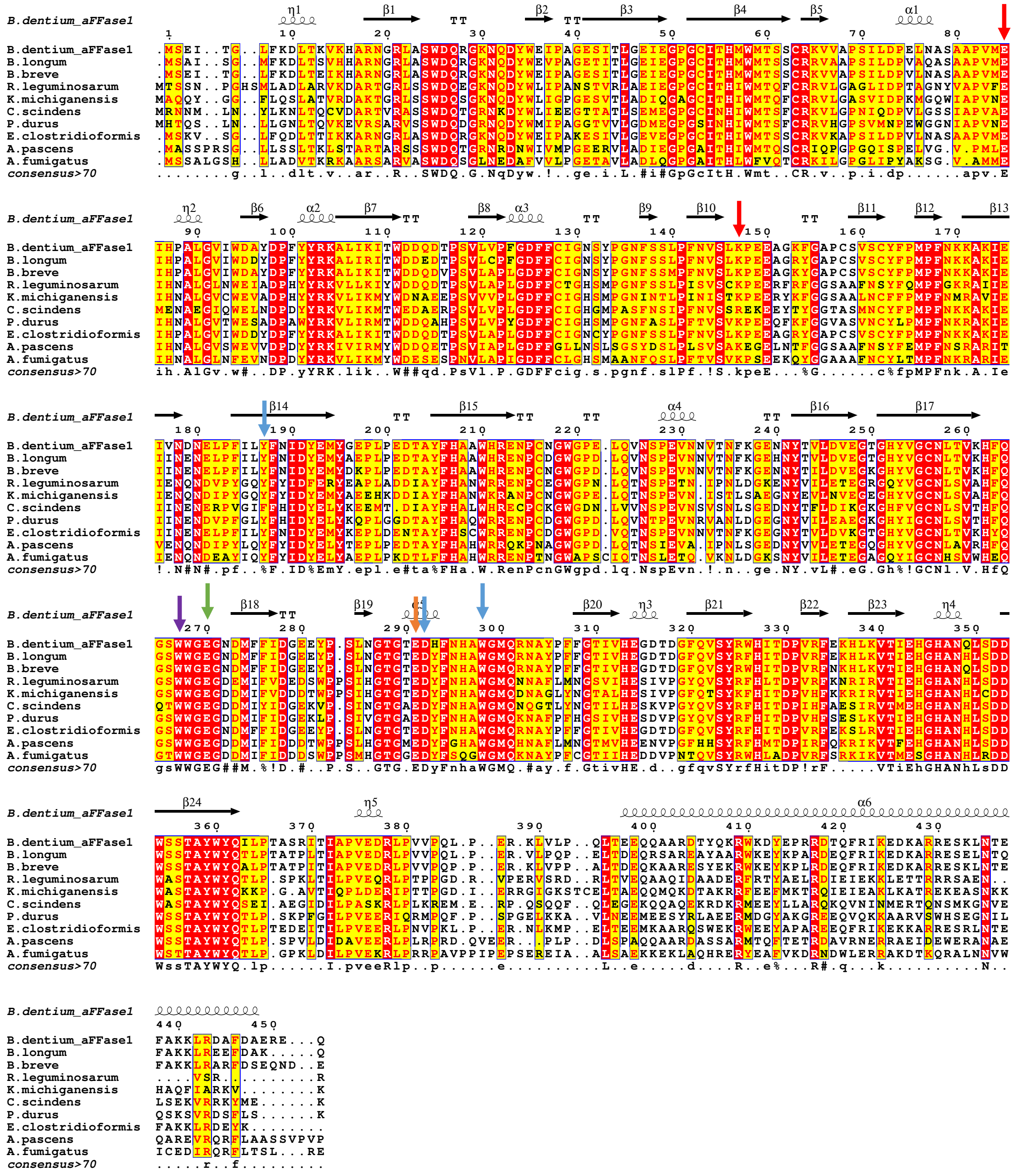

Figure S16. Multiple amino acid sequence alignment of αFFase1 and selected homologs. The protein sequences were selected from the phylogenetic tree in Fig. S15. Residues constituting the −1 and +1 subsites are indicated by blue and red arrows, respectively. W267, which forms both −1 and +1 subsites, is indicated by a purple arrow. E291 (nucleophile) and E270 (acid/base catalyst) are indicated by orange and green arrows, respectively. ESPript 3.0 was used. GenBank accession numbers are as follows: *B. longum*, WP_14054715.1; *B. breve*, WP_021649236.1; *R. leguminosarum*, WP_116272183.1; *K. michiganensis*, WP_049101457.1; *C. scindens*, WP_004604830.1; *P. durus*, WP_025696556.1; *E. clostridioformis*; WP_027644080.1; *A. pascens*, WP_205651602.1; *A. fumigatus*, XP_748288.1.

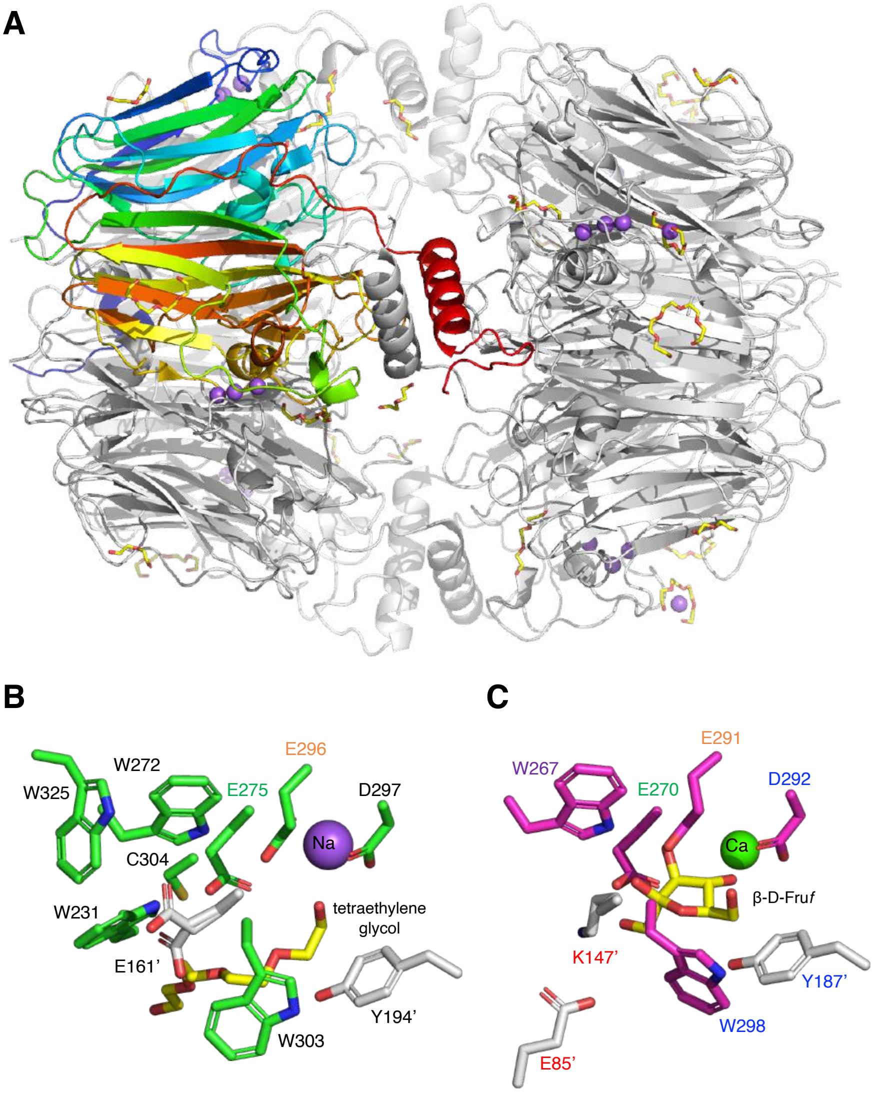

Figure S17. Structural comparison with BACUNI_00161. *A*, Hexameric structure of BACUNI_00161 generated by crystallographic symmetry mates. One protomer is colored in blue at the N-terminus to red at the C-terminus. Bound polyethylene glycol molecules and sodium ions are shown as yellow sticks and purple spheres, respectively. *B*, Putative active site of BACUNI_00161. *C*, The active site of αFFase1 for comparison. Residues from a neighboring protomer are shown as white sticks.

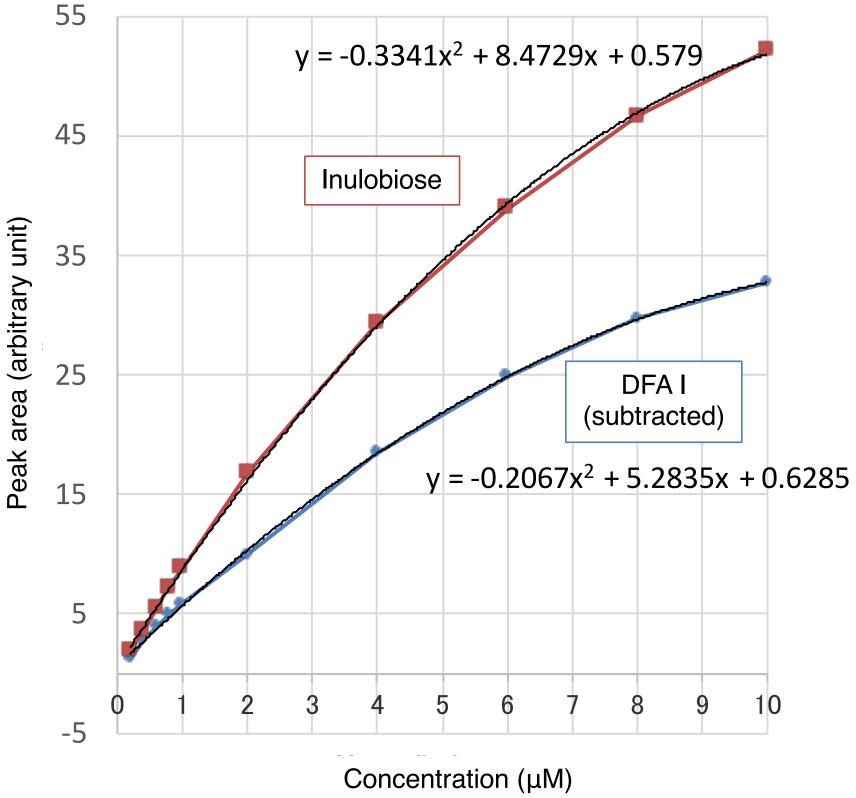

Figure S18. Calibration curves for detection of DFA I and inulobiose by HPAEC-PAD. Colored plots are measurements of standard samples. The equations were calculated form the fitting curves (black lines) and were used for calculating concentration of the compounds.

Supplementary Movie S1. Movie of inulobiose in the active site of chain A during the 1 µs MD run. The ligand and water molecules within 5 Å are shown at intervals of 100 ns.

Supplementary Movie S2. Movie of DFA I in the active site of chain B during the 1 µs MD run. The ligand and water molecules within 5 Å are shown at intervals of 100 ns.

Table S1. Chemical shifts and *J* coupling constants of the peracetylated peak (3) by ^1H and ^{13}C NMR analysis

	^1H	^3J Hz	^{13}C	Key HMBC
**Fru*p***				
**1**	3.71	d, 12.0	61.12	→ 101.54 (Fru*f*-C2), 94.99 (Fru*p*-C2)
	3.82	d, 12.0		
**2**	-	-	94.99	
**3 (Ac)**	3.56	d, 13.2	67.30	
**4 (Ac)**	5.37	dd, 10.0, 3.2	67.51	
**5 (Ac)**	5.33	M	68.95	
**6**	3.79	dd, 13.2, 1.6	61.54	→ 94.99 (Fru*p*-C2)
	3.84	d, 13.2		
**Fru*f***				
**1**	3.55	d, 12.0	61.54	→ 101.54 (Fru*f*-C2)
	3.85	d, 12.0		
**2**	-	-	101.54	
**3 (Ac)**	5.18	d, 2.0	81.23	
**4 (Ac)**	4.94	dd, 5.6, 2.0	77.86	
**5**	4.16	td, 5.6, 3.2	79.65	
**6 (Ac)**	4.21	dd, 12.0, 3.2	63.19	
	4.38	dd, 12.0, 5.6		
**Ac**	2.14, 2.11, 2.09, 2.07, 2.05, 1.96	s	170.46 (x2), 170.35, 170.04, 169.01v (x2), 20.95, 20.74 (x2), 20.68, 20.61 (x2)	

Table S2. Crystallographic data collection and refinement statistics of αFFase1

**Data set**	**Ligand free**	**D-Arabinose**	**D-Fructose**
Data collection [a]			
Beamline	KEK PF BL-5A	SLS X06SA	SLS X06SA
Wavelength (Å)	1.0000	1.0000	1.0000
Space group	$P2_1$	$P2_1$	$P2_1$
Unit cell			
a, b, c (Å)	98.55, 156.97, 100.91	98.45, 156.71, 100.85	98.44, 156.30, 100.74
β (°)	109.93	110.66	110.09
Resolution (Å)	49.64–1.96	48.72–1.86	48.64–1.76
	(1.99–1.96)	(1.90–1.86)	(1.79–1.76)
Total reflections	716,504 (33025)	1,638,158 (78855)	1,948,150 (89388)
Unique reflections	206,183 (10,181)	237,104 (11,654)	279,590 (13,723)
$CC_{1/2}$	0.99 (0.80)	1.00 (0.81)	1.00 (0.78)
Completeness (%)	99.9 (99.9)	100.0 (100.0)	99.3 (98.4)
Multiplicity	3.5 (3.3)	6.9 (6.8)	7.0 (6.5)
Mean $I/\sigma(I)$	10.3 (2.9)	10.9 (2.4)	11.8 (2.4)
R_{merge}	0.098 (0.432)	0.113 (0.774)	0.107 (0.729)
Refinement			
Resolution (Å)	49.64–1.96	48.77–1.86	48.69–1.76
No. of reflections	205,845	237,064	279,569
R_{work}/R_{free} [b]	0.150/0.190	0.171/0.212	0.158/0.195
Number of atoms	24,209	23,591	23,998
Amino acids	21,732	21,768	21,768
Ions	6	6	6
Ligands	36	60	72
Waters	2,423	1,752	2,139
B-factors (Å^2)			
Amino acids	18.7	28.5	22.0
Ions	13.6	14.5	10.3
Ligands	44.4	26.8	23.6
Waters	25.3	31.67	28.7
RMSD from ideal values			
Bond lengths (Å)	0.010	0.009	0.011

Bond angles (°)	1.617	1.593	1.681
Ramachandran plot (%)			
Favored	95.6	95.3	95.4
Allowed	4.1	4.4	4.3
Outlier	0.3	0.3	0.3
PDB code	7V1V	7V1W	7V1X

[a] Values in parentheses are for the highest resolution shell.

[b] R_{free} was calculated for a randomly chosen 5% of reflections, which were not used for structure refinement, and R_{work} is calculated for the remaining reflections.

Table S3. Results of the structural similarity search using the Dali server

Protein	Source organism	PDB (chain)	Z score	RMSD (Å)	N_{align}[a]	$\%_{seq}$[b]
Full length (3-449)						
Hypothetical protein BACUNI_00161	*Bacteroides uniformis*	4KQ7 (A)	42.3	2.0	335	36
Coat protein	*Sulfolobus* turreted icosahedral virus 1	2BBD (B)	13.9	4.0	249	7
Major capsid protein	*Enterobacteria* phage PRD1	1GW7 (A)	12.7	4.7	243	4
Major capsid protein P2	*Pseudoalteromonas* virus PM2	2W0C (C)	12.1	3.8	225	8
Major capsid protein	Bacteriophage sp.	5OAC (H)	10.3	4.4	222	8
Major capsid protein	Faustovirus	5J7O (F)	9.0	4.8	239	9
Major capsid protein	*Paramecium bursaria* Chlorella virus 1	5TIQ (B)	8.4	4.7	241	8
Rifampicin resistance protein	Vaccinia virus WR	3SAM (C)	8.2	5.0	238	5
Major capsid protein	Singapore grouper iridovirus	6OJN (B)	8.1	5.6	230	7
β-jelly roll 1 (3-200)						
Hypothetical protein BACUNI_00161	*Bacteroides uniformis*	4KQ7 (A)	20.2	1.5	154	34
VP1	*Haloarcula hispanica* icosahedral virus 2	6H82 (B)	8.9	2.9	108	12
C381 turret protein	*Sulfolobus* turreted icosahedral virus 1	4IND (N)	8.8	6.6	109	7
Tripeptidyl-peptidase 2	*Drosophila melanogaster*	3LXU (X)	8.4	3.5	111	6
Transmembrane EMP24 domain-containing protein 10	*Mus musculus*	5GU5 (A)	8.1	2.5	93	10
GH84 *O*-GlcNAcase	*Bacteroides thetaiotamicron*	5FL1 (A)	6.5	4.4	95	13
CBM61	*Thermotoga maritima*	2XOM (A)	6.5	3.2	105	9
β-jelly roll 2 (201-390)						

Hypothetical protein BACUNI_00161	*Bacteroides uniformis*	4KQ7 (A)	25.5	2.2	180	39
GH86 β-agarase BuGH86	*Bacteroides uniformis*	5TA1 (A)	5.6	4.0	108	627

[a] Number of aligned residues.

[b] Sequence identity.

Table S4. Sugar ring conformations observed in the −1 subsite of αFFase1 as analyzed by Altona-Sundaralingam parameters.

Furanose	PDB ID	Chain	Conformation	*P* (°)	φ_m (°)
β-D-Ara*f*	7V1W	A	$^3T_4$	40.02	35.78
		B	$^3T_4$	37.33	31.57
		C	$^3T_4$	39.03	35.92
		D	$^3T_4$	29.95	31.51
		E	$^3T_4$	35.56	31.96
		F	$^3T_4$	30.06	35.36
			(Average ± SD)	35.3 ± 4.0	33.7 ± 2.0
β-D-Fru*f*	7V1X	A	E_5	53.12	39.66
		B	E_5	55.43	41.24
		C	E_5	51.89	39.69
		D	E_5	51.45	38.35
		E	E_5	52.35	41.25
		F	E_5	59.18	39.81
			(Average ± SD)	53.9 ± 2.7	39.9 ± 1.0

SD: standard deviation.

Table S5. Statistics of MD analysis

Model	Chain	RMSD (Å)[a]			No. of waters[b]
		**Protein**	**Ca^{2+}**	**Ligand[c]**	
β-D-Fru*f*	A	1.00 ± 0.09	0.78 ± 0.18	2.12 ± 0.68*	7.75 ± 1.94
	B	1.10 ± 0.11	1.83 ± 0.25	1.31 ± 0.25*	7.37 ± 1.40
	C	1.05 ± 0.10	1.58 ± 0.55	18.60 ± 19.75**	8.87 ± 1.94
	D	1.22 ± 0.18	1.32 ± 0.37	1.53 ± 0.55*	8.38 ± 1.36
	E	0.98 ± 0.08	1.45 ± 0.50	1.20 ± 0.25*	8.70 ± 1.76
	F	1.18 ± 0.14	0.81 ± 0.21	1.23 ± 0.33*	8.80 ± 1.53
Inulobiose	A	1.03 ± 0.10	1.98 ± 0.42	2.35 ± 0.26	8.82 ± 1.67
	B	1.09 ± 0.19	1.77 ± 0.60	2.45 ± 0.22	8.43 ± 1.65
	C	1.18 ± 0.12	1.60 ± 0.48	2.34 ± 0.36	8.56 ± 1.52
	D	1.05 ± 0.11	1.56 ± 0.61	2.50 ± 0.25*	8.99 ± 1.73
	E	1.15 ± 0.12	2.08 ± 0.33	2.13 ± 0.26	8.90 ± 1.54
	F	1.13 ± 0.13	1.32 ± 0.60	3.13 ± 0.91*	7.86 ± 1.97
DFA I	A	1.07 ± 0.10	1.97 ± 0.40	2.43 ± 0.59*	7.75 ± 1.94
	B	1.11 ± 0.11	2.03 ± 0.39	2.14 ± 0.21	7.37 ± 1.40
	C	1.10 ± 0.10	0.95 ± 0.23	3.83 ± 0.44*	8.87 ± 1.94
	D	1.10 ± 0.10	1.99 ± 0.32	2.16 ± 0.21	8.38 ± 1.36
	E	1.08 ± 0.15	1.57 ± 0.47	4.02 ± 0.70*	8.70 ± 1.76
	F	1.04 ± 0.07	2.09 ± 0.49	2.22 ± 0.28	7.80 ± 1.53

The average and standard deviation values during the 1 μs MD run were calculated using the snapshot structures every 1 ns.

[a] Root mean square deviation of each snapshot structure from the initial state was calculated for the Cα atoms (protein), the Ca atom (Ca^{2+}), and the non-hydrogen atoms (ligand) after aligning the Cα atoms of the snapshot structure to those of the initial structure.

[b] Number of water molecules with oxygen atoms within 3.5 Å from the ligand oxygen atoms.

[c] Asterisks indicate that the ligand molecule moved in the active site pocket (single asterisk) or moved out of the pocket (double asterisks).

Table S6. BBDE_2040 and BBDE_2039 homologs in draft genomes of *B. breve* strains isolated from human feces

**Strain[a]**	**BBDE_2040 homolog**	**BBDE_2039 homolog**
MCC 0121 (infant)	BBM0121_06860	BBM0121_06855
MCC 0476 (adult)	BBM0476_07465	BBM0476_07470
MCC 1114 (infant)	BBM1114_09685	BBM1114_09690
MCC 1128 (infant)	BBM1128_00740	BBM1128_00745
MCC 1340 (infant)	BBM1340_10310	BBM1340_10305
MCC 1605 (elderly)	BBM1605_09080	BBM1605_09075

Among the ten *B. breve* strains studied by Odamaki et al. (1), the genomes of the six strains contained homologs of *BBDE_2040* and *BBDE_2039*. Sequence identities of all homologs with BBDE_2040 and BBDE_2039 were 91.4 and 76.7%, respectively. Sequence identities were calculated using the EMBOSS Needle (2).

[a] Age category of the isolation source individual is shown in parentheses.

Table S7. Primers used for gene cloning and site-directed mutagenesis of αFFase1

Object	Sequence
BBDE_2040 amplification	5'-AGGAGATATACCATGAGTGAAATCACTGGTC-3'<br>3'-CTACGCCTCTCTCTTGTTGAGTCGTGGTGGTG-5'
pET23d amplification	5'-CTCGAGCACCACCACCACCACCACTG-3'<br>3'-TTGAAATTCTTCCTCTATATGGTAC-5'
E85A mutation	5'-GCTCCGGTGATGGCGATTCACCCGGCA-3'<br>5'-CGAGGCCACTACCGCTAAGTGGGCCGT-3'
E85Q mutation	5'-GGCTCCGGTGATGCAGATTCACCCGGC-3'<br>3'-CCGAGGCCACTACGTCTAAGTGGGCCG-5'
K147A mutation	5'-CCGTTTAATGTATCGCTCGCGCCGGAGGAAGCAGGAAA-3'<br>3'-GGCAAATTACATAGCGAGCGCGGCCTCCTTCGTCCTTT-5'
W267A mutation	5'-GCACTTCCAAGGAAGTGCGTGGGGAGAAGGCAAC-3'<br>3'-GTTGCCTTCTCCCCACGCACTTCCTTGGAAGTGC-5'
Y187A mutation	5'-TAACGAGTTGCCGTTCATTCTGGCCTTCAACATCGACTATGAAATG-3'<br>3'-ATTGCTCAACGGCAAGTAAGACCGGAAGTTGTAGCTGATACTTTAC-5'
Y187F mutation	5'-GAGTTGCCGTTCATTCTGTTCTTCAACATCGACTATGAA-3'<br>3'-CTCAACGGCAAGTAAGACAAGAAGTTGTAGCTGATACTT-5'
E270A mutation	5'-GGAAGTTGGTGGGGAGCAGGCAACGATATGTTC-3'<br>3'-CCTTCAACCACCCCTCGTCCGTTGCTATACAAG-5'
E270Q mutation	5'-CCAAGGAAGTTGGTGGGGACAGGGCAACGATATGTTC-3'<br>3'-GGTTCCTTCAACCACCCCTGTCCCGTTGCTATACAAG-5'
E291A mutation	5'-AACGGCACCGGTACTGCGGATTATTTCAACCATG-3'<br>3'-TTGCCGTGGCCATGACGCCTAATAAAGTTGGTAC-5'
E291Q mutation	5'-GAGTTTGAACGGCACCGGTACTCAGGATTATTTCAA-3'<br>3'-CTCAAACTTGCCGTGGCCATGAGTCCTAATAAAGTT-5'
D292A mutation	5'-GGCACCGGTACTGAGGCTTATTTCAACCATGCC-3'<br>3'-CCGTGGCCATGACTCCGAATAAAGTTGGTACGG-5'
D292N mutation	5'-ACGGCACCGGTACTGAGAATTATTTCAACCATGCC-3'<br>3'-TGCCGTGGCCATGACTCTTAATAAAGTTGGTACGG-5'
W298A mutation	5'-GATTATTTCAACCATGCCGCGGGTATGCAGCGCAATGC-3'<br>3'-CTAATAAAGTTGGTACGGCGCCCATACGTCGCGTTACG-5'

References

1. Odamaki, T., Horigome, A., Sugahara, H., Hashikura, N., Minami, J., Xiao, J. Z., and Abe, F. (2015) Comparative Genomics Revealed Genetic Diversity and Species/Strain-Level Differences in Carbohydrate Metabolism of Three Probiotic Bifidobacterial Species. *Int. J. Genomics*. 10.1155/2015/567809
2. Needleman, S. B., and Wunsch, C. D. (1970) A general method applicable to the search for similarities in the amino acid sequence of two proteins. *J. Mol. Biol.* **48**, 443–453
